# Angiocrine signals regulate quiescence and therapy resistance in bone metastasis

**DOI:** 10.1172/jci.insight.125679

**Published:** 2019-07-11

**Authors:** Amit Singh, Vimal Veeriah, Pengjun Xi, Rossella Labella, Junyu Chen, Sara G. Romeo, Saravana K. Ramasamy, Anjali P. Kusumbe

**Affiliations:** 1 The Kennedy Institute of Rheumatology, University of Oxford, Oxford, United Kingdom.; 2 Department of Prosthodontics, West China Hospital of Stomatology, Sichuan University, Chengdu, China.; 3 Institute of Clinical Sciences, Imperial College London, London, United Kingdom.; 4 MRC London Institute of Medical Sciences, Imperial College London, London, United Kingdom.

**Keywords:** Bone Biology, Vascular Biology, Adult stem cells, Bone marrow, Cancer

## Abstract

Bone provides supportive microenvironments for hematopoietic stem cells (HSCs) and mesenchymal stem cells (MSCs) and is a frequent site of metastasis. While incidences of bone metastases increase with age, the properties of the bone marrow microenvironment that regulate dormancy and reactivation of disseminated tumor cells (DTCs) remain poorly understood. Here, we elucidate the age-associated changes in the bone secretome that trigger proliferation of HSCs, MSCs, and DTCs in the aging bone marrow microenvironment. Remarkably, a bone-specific mechanism involving expansion of pericytes and induction of quiescence-promoting secretome rendered this proliferative microenvironment resistant to radiation and chemotherapy. This bone-specific expansion of pericytes was triggered by an increase in PDGF signaling via remodeling of specialized type H blood vessels in response to therapy. The decline in bone marrow pericytes upon aging provides an explanation for loss of quiescence and expansion of cancer cells in the aged bone marrow microenvironment. Manipulation of blood flow — specifically, reduced blood flow — inhibited pericyte expansion, regulated endothelial PDGF-B expression, and rendered bone metastatic cancer cells susceptible to radiation and chemotherapy. Thus, our study provides a framework to recognize bone marrow vascular niches in age-associated increases in metastasis and to target angiocrine signals in therapeutic strategies to manage bone metastasis.

## Abstract 2

Radiation and chemotherapy induce bone-specific expansion of vascular niches, which promotes therapy resistance.

## Graphical abstract

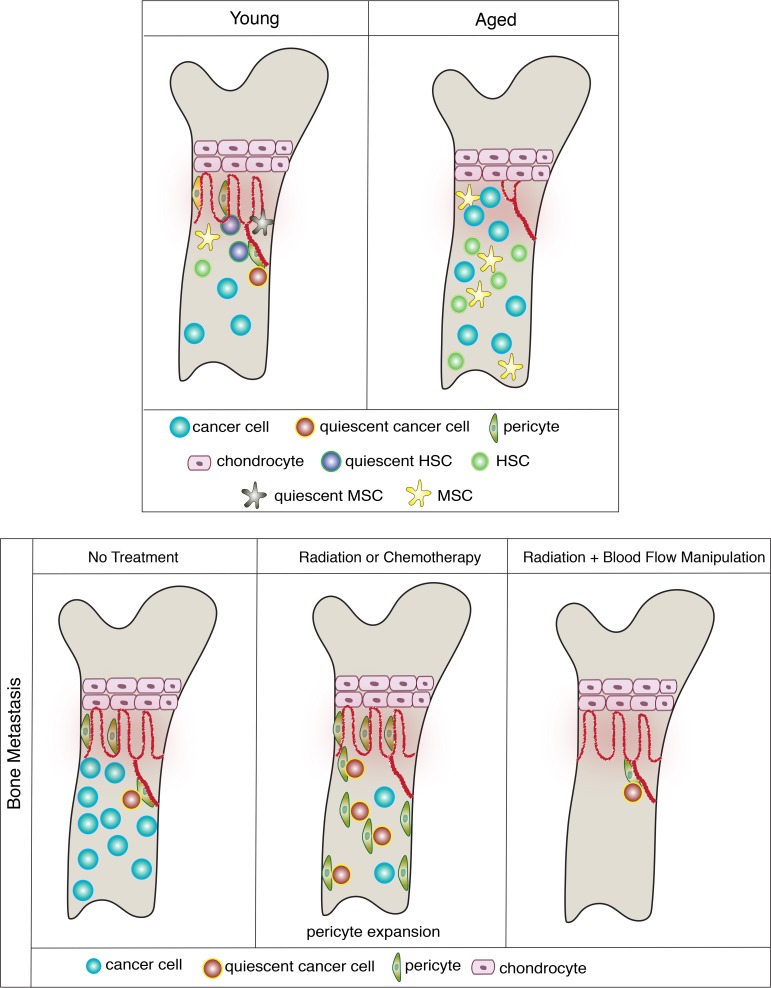

## Introduction

In addition to supplying oxygen and nutrients, the vasculature provides a number of inductive factors and signals, so-called angiocrine signals, to regulate tissue functions. In the skeletal system, blood vessels play a central role in the maintenance of microenvironment required for regulating osteogenesis and hematopoiesis (1–7). Blood vessels also regulate aging of the hematopoietic and skeletal systems. Specifically, skeletal aging is associated with the decline in tissue homeostasis, decreased osteogenesis, and altered hematopoiesis (8–10). Hematopoietic stem cells (HSCs) exhibit age-associated structural and functional alterations that involve skewing toward myeloid lineage and loss of reconstitution potential (11–13). Similarly, bone marrow (BM) mesenchymal stem cells (MSCs) are thought to undergo age-related changes and exhibit a bias toward an adipogenic lineage (14, 15). Cell-intrinsic mechanisms have been reported to regulate aging of HSCs, MSCs, and other BM cellular compartments (11, 14, 16, 17). However, the contribution of cell-extrinsic signals and the impact of the BM microenvironment toward stem cell aging and quiescence remain poorly understood (18–20).

Aging is also linked to increased incidences of metastatic relapse, with bone as one of the most frequent site of metastasis (20–24). The dissemination of tumor cells to bone precedes primary tumor detection (25–27). Consequently, the therapeutic regimens used to target the primary tumor are likely to impact the behavior of disseminated tumor cells (DTCs) (28). However, at least a fraction of DTCs escape therapeutic regimens and survive within the BM microenvironment in a quiescent state for a very long period, with a subset of patients developing detectable metastases as late as 2–3 decades after detection of the primary tumor (29–31). Such clinical evidence argues in favor of targeting DTCs within the bone microenvironment instead of targeting the initial steps of extravasation and dissemination (24, 29–34). Designing strategies to target quiescent DTCs in bone that escape conventional therapies during primary tumor treatment will facilitate the development of therapeutic regimens to prevent metastatic relapse in bone (35, 36). However, this requires the fundamental understanding of niches and signals that support cellular quiescence in the BM and changes induced by aging and therapies. Here, we interrogate and define the cues in the BM microenvironment that impact stem and cancer cell quiescence, proliferation, and chemoresistance. We also investigate age-dependent and therapy-induced alterations in the BM microenvironment.

## Results

### Niche-dependent perturbation of stem and cancer cell quiescence during aging.

**Figure 1:**
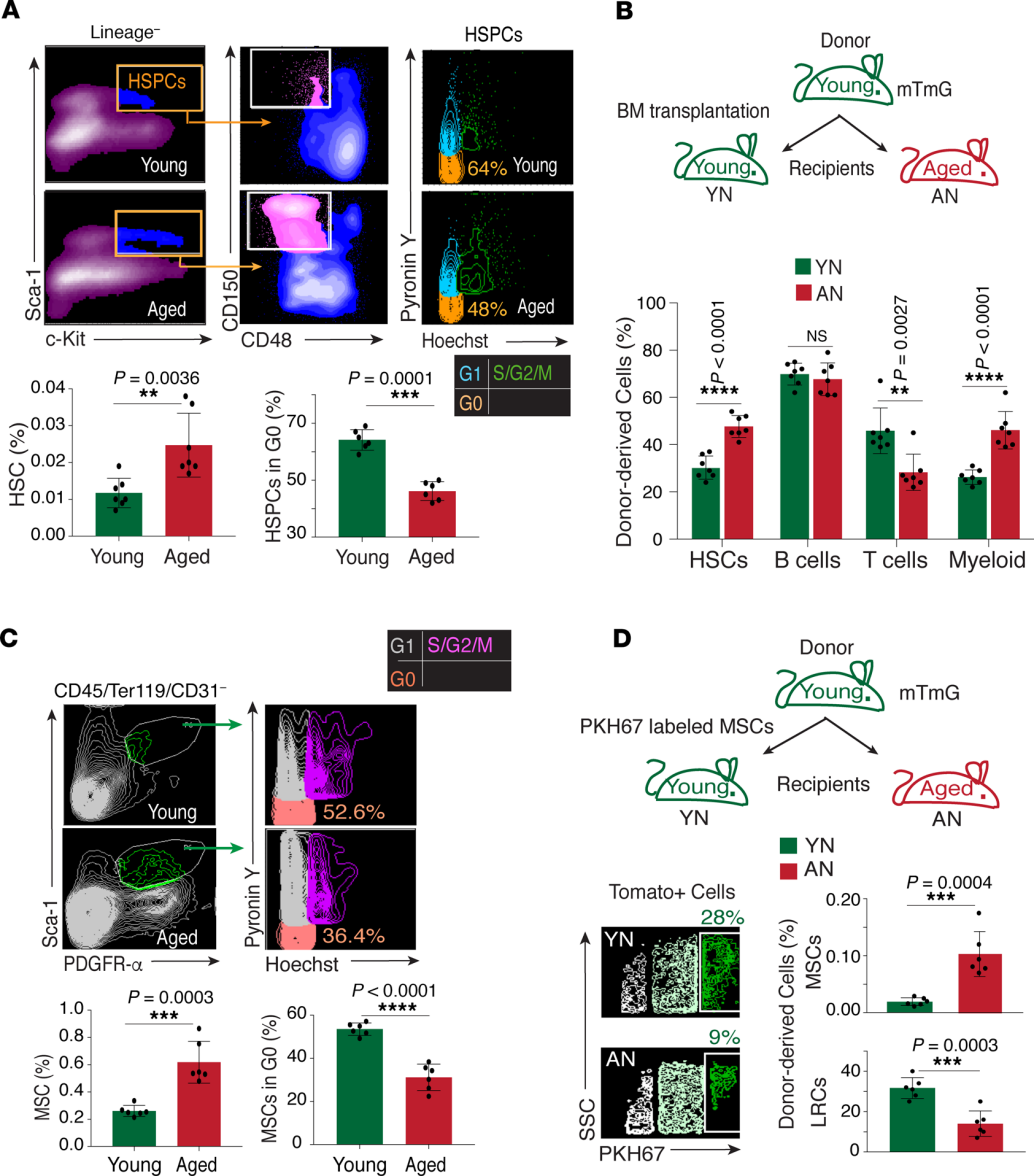
Niche-dependent perturbation of HSC and MSC quiescence in aging. (**A**) FACS quantification (as shown in contour plots) of HSCs in young (*n* = 7 replicates) and aged (*n* = 7 replicates ) long bones (left panel). Data represent mean ± SD; 2-tailed unpaired *t* test. G_0_ and G_1_ phases separated by staining with Pyronin Y, which preferentially binds to RNA, and Hoechst 33342, which in turn binds to A-T base pairs. The lower right panel shows flow cytometric quantification of quiescent HSPCs. Data represent mean ± SD (*n* = 6 replicates); 2-tailed unpaired *t* test. (**B**) Schematic representation of the experimental strategy. The graph shows the frequency of the young Tomato**^+^** donor-derived HSCs, B cells, T cells, and myeloid cells in the BM of the young and aged recipients after 16 weeks following transplantation. Data represent mean ± SD (*n* = 7 replicates); 2-tailed unpaired *t* test. YN, young niche, determined by quantification in bones from young mice; AN, aged niche, determined by quantification in bones from aged mice. (**C**) Representative FACS plots from young and aged mouse bones show the gating strategy for identifying quiescent (G_0_) MSCs, which is based on Hoechst and Pyronin Y staining. The lower left panel shows FACS quantification of MSCs based on PDGFRα^+^Sca-1^+^CD45^–^Ter119^–^CD31^–^ expression in the young and aged mice. The lower right panel shows the quantification of quiescent (G_0_) MSCs in young and aged bones based on Hoechst and Pyronin Y staining. Data represent mean ± SD (*n* = 6 replicates); 2-tailed unpaired *t* tests. (**D**) Schematic illustration of the transplantation strategy for Tomato^+^ PKH67^+^ MSCs in the young and aged recipients. The upper bar graph shows FACS-based quantification of total Tomato^+^ donor PDGFRα^+^Sca-1^+^CD45^–^Ter119^–^CD31^–^ MSCs in young and aged mouse bones 10 weeks after transplantation. The bar graph in the lower panel shows the quantification of label retaining the MSCs 10-weeks post transplantation. The data represent the mean ± SD (*n* = 6 replicates), and 2-tailed unpaired *t* tests. **P < 0.01, ***P < 0.001, ****P < 0.0001.

HSCs show reduced regenerative and functional potential with age, whereas their number increases upon aging (11, 37, 38). In line with the previous reports, we found that the CD150^+^CD48^–^Lin^–^Sca-1^+^c-Kit^+^ HSC fraction increased in aged mice as compared with their young counterparts. Quantification of cells in the G_0_ state within the hematopoietic stem progenitor cell (HSPC) fraction (Lin^–^Sca-1^+^c-Kit^+^) using Hoechst and Pyronin Y staining suggested a decline in the quiescent population with age (Figure 1A). To understand the contribution of the microenvironment in regulating the age-dependent phenotype of HSCs, we performed heterochronic transplant assays in which BM cells from young donors were transplanted into both young and aged recipients (Figure 1B). Posttransplantation analysis at 16 weeks showed that aged recipients demonstrate an elevated frequency of HSCs and myeloid cells, while the frequency of T cells was decreased, and B cells remained unaltered (Figure 1B). Next, we analyzed the age-dependent changes in the MSC compartment residing within the BM. Similar to the HSCs, the frequency of PDGFRα^+^Sca-1^+^CD45^–^Ter119^–^CD31^–^ MSCs in bones demonstrated an age-dependent increase, whereas the frequency of quiescent subset (MSCs in the G_0_ state) declined (Figure 1C). We performed the heterochronic transplant assays with MSCs in which purified Tomato^+^ MSCs from young mice were labeled with PKH67 (to identify label-retaining cells [LRCs]) and then transplanted into young and aged recipients (Figure 1D). In comparison to young recipients, transplanted MSCs expanded at a higher frequency in aged mice as determined by the quantification of Tomato^+^ PDGFRα^+^ Sca-1^+^CD45^–^Ter119^–^CD31^–^ MSCs in recipient bones. However, levels of LRCs or quiescent cells, as determined by retention of the membrane dye PKH67 within this MSC fraction, declined significantly in aged recipients (Figure 1D). Collectively, the above findings support the important contribution of the microenvironment in regulating age-dependent changes in phenotypes and quiescence of HSCs and MSCs in bones.

**Figure 2:**
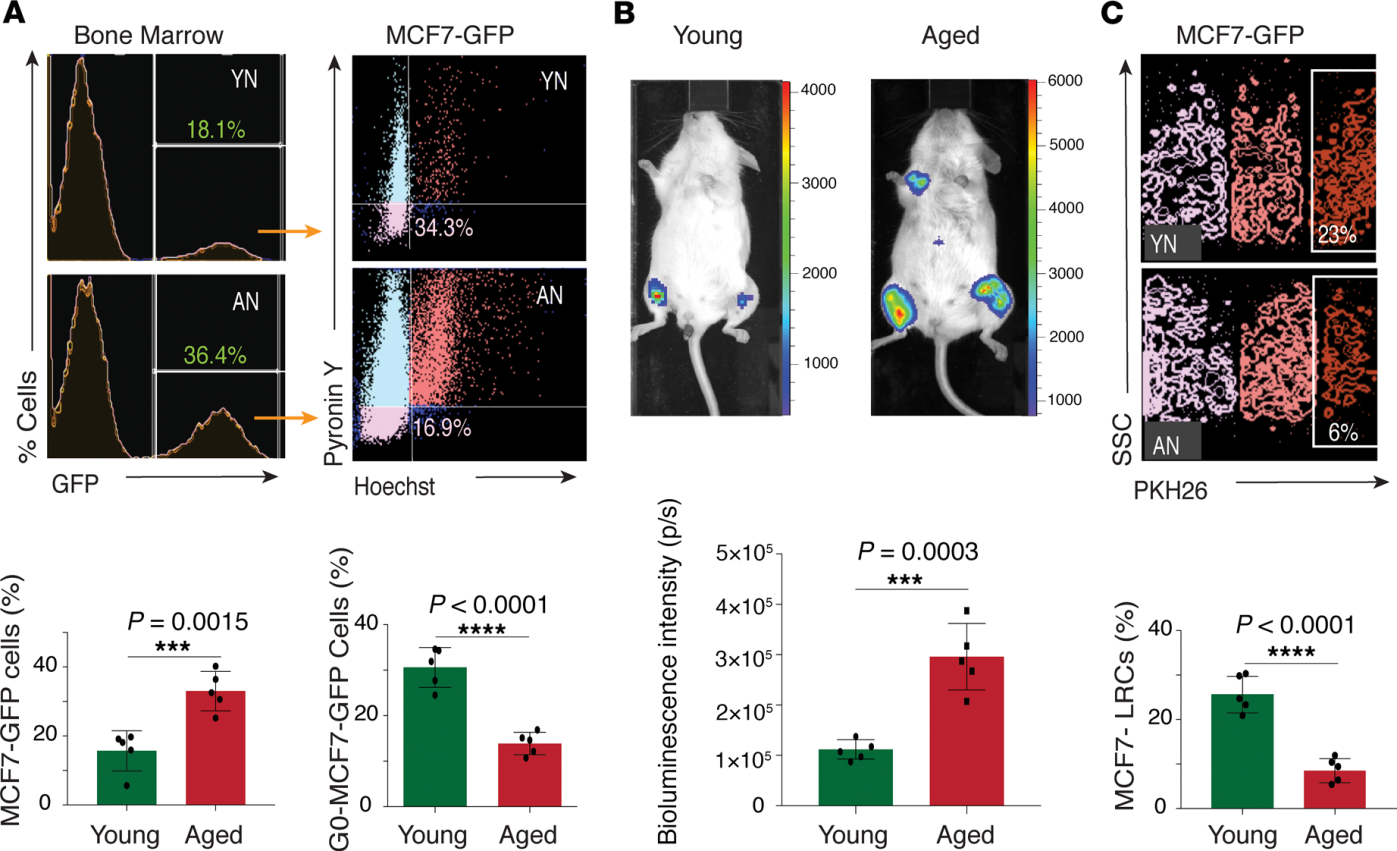
Bone metastatic cells exhibit a decline in quiescence in the aged BM microenvironment. (**A**) Representative FACS histogram plots show MCF7-GFP cells (top left) in young and aged mouse bones 19 days after intratibial injection. FACS plots in the right panel show Hoechst and Pyronin Y staining on the gated MCF7 cells. The left bar graph shows the flow cytometric quantification of MCF7-GFP cells in the single-cell suspension of the young and aged mouse bones. The right bar graph demonstrates the quantification of quiescent (G_0_) MCF7-GFP cells in the bones of young and aged mice after the intratibial injections. Data represent mean ± SD (*n* = 5 replicates); 2-tailed unpaired *t* tests. YN, young niche, determined by quantifications in bones from young mice; AN, aged niche, determined by quantifications in bones from aged mice. (**B**) Bioluminescence analysis of the representative mouse from the young and aged groups 3 weeks after the intracardiac injections. The color bar indicates luminescence counts. The graph shows bioluminescent intensity in the young and aged mice. Data represent mean ± SD (*n* = 5 replicates); 2-tailed unpaired *t* test. (**C**) Representative FACS contour plots show the gating strategy for identifying the label retaining the PKH26^hi^ fraction of the MCF7-GFP cells in single-cell suspensions of the young and aged mouse bones. The graph shows the quantification of the label retaining the MCF7-GFP cells in aged and young tibiae. Data represent the mean ± SD (*n* = 5 replicates); 2-tailed unpaired *t* test. ***P < 0.001; ****P < 0.0001.

The skeleton being a dynamic microenvironment and a frequent site for metastasis, we investigated how age-related changes in the BM microenvironment impact cancer cell behavior in bones. To investigate this, we analyzed the expansion, proliferation, and quiescence of bone metastatic cancer cells in young and aged mice. Specifically, the human breast cancer cell lines MDA-MB-231, MCF7, and ZR-75-1 were used to generate bone metastasis by intratibial or intracardiac injections. Analogous to HSCs and MSCs, these bone metastatic cell lines were highly proliferative in the aged bone microenvironment, which led to significant expansion of these cells in aged mice when compared with young mice (Figure 2, A and B, and Supplemental Figure 1, A and B; supplemental material available online with this article; https://doi.org/10.1172/jci.insight.125679DS1). Analysis of quiescent fraction of cancer cells in the BM, as determined by a G_0_ state (Hoechst and Pyronin Y staining) and label retention assay (PKH26 membrane labeling), revealed that the frequency of quiescent bone metastatic cancer cells declined in aged mice (Figure 2, A and C, and Supplemental Figure 1, C and D). Quiescent cancer cells have been shown to resist chemotherapy and radiotherapy. These results, demonstrating the proliferation-promoting nature of the aging BM microenvironment, provide a plausible explanation for metastatic relapse observed in some patients after long (up to several decades) dormancy periods.

### Downregulation of quiescence-inducing factors in the aged BM microenvironment.

**Figure 3:**
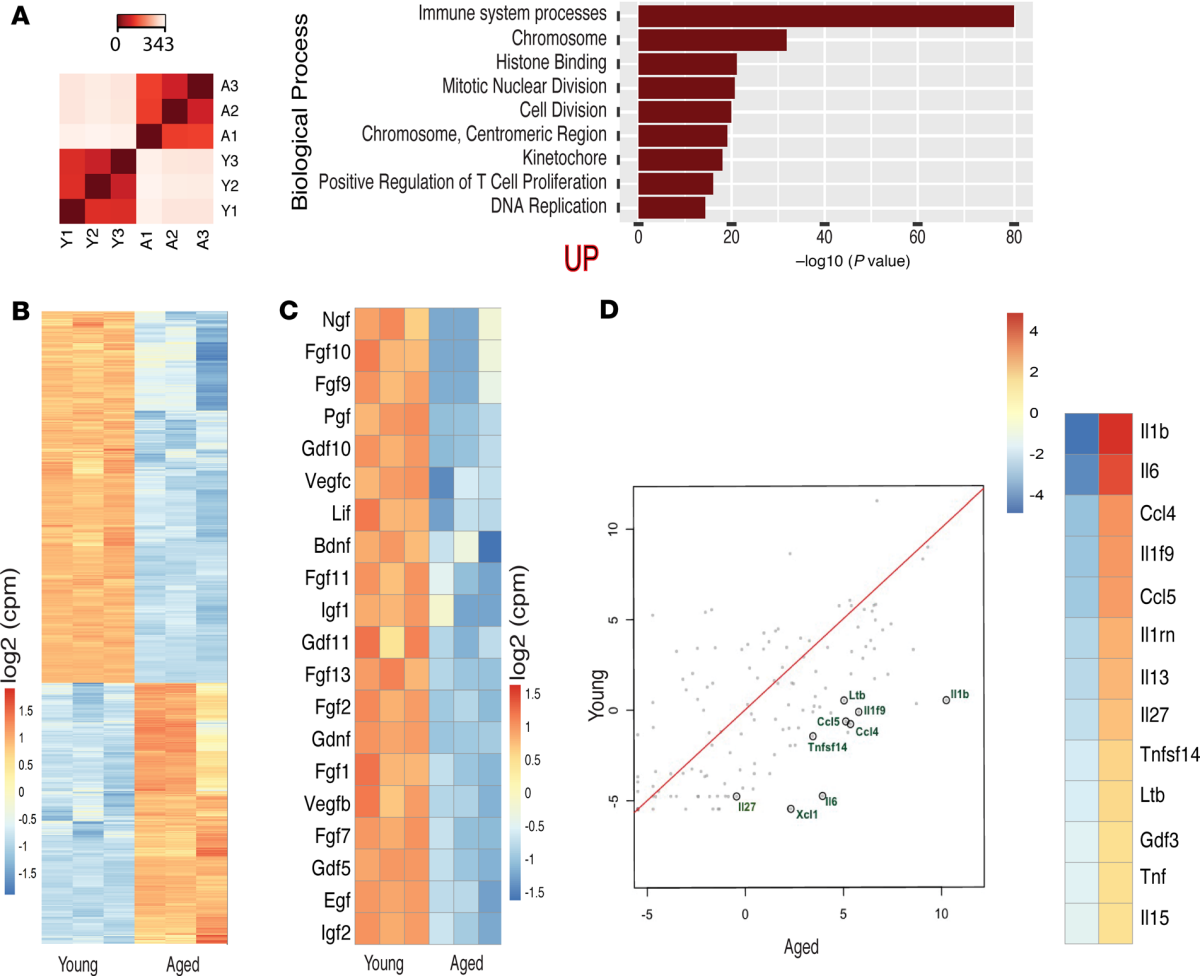
Multiple cytokines and growth factors are altered in the aged BM. (**A**) The heatmap represents hierarchical clustering of sample-to-sample distance to assess the data quality by sample-to-sample distance clustering. Variance-stabilizing transformation of the RNA-Seq read count for all the samples was used to calculate the sample-to-sample Euclidean distance (color scale) for hierarchical clustering, i.e., young (Y1–Y3), aged (A1–A3). Gene set enrichment analysis (GSEA) of young and aged long bones was performed. The most significant biological processes were assessed by GAGE, with *q* < 0.000001. The top differentially upregulated (UP) biological processes are shown in the bar graph. The *y* axis shows the GO terms, and the *x* axis represents the enrichment scores of these terms. (**B**) The heatmap shows 7281 differentially expressed genes between the young and aged bones (FDR-adjusted *P* value cutoff <0.01, log_2_ fold change ±1). The color intensity indicates the row-scaled-normalized log_2_(cpm) expression values. The columns display the data for each of the 3 replicates. (**C**) The heatmap shows the most significant growth factors that are differentially expressed in the young in comparison to the aged bones/BM. The color code indicates the row mean subtracted from the normalized log_2_(cpm) expression values. (**D**) The scatterplot shows the average normalized log_2_(cpm) aged bones versus the average normalized log_2_(cpm) young bones. The cytokines highlighted in black circles are significantly upregulated in the aged BM. The red line indicates the slope and the intercept. The heatmap shows the most significantly differentially expressed cytokines in the young (left column) versus aged (right column) bones.

**Figure 4:**
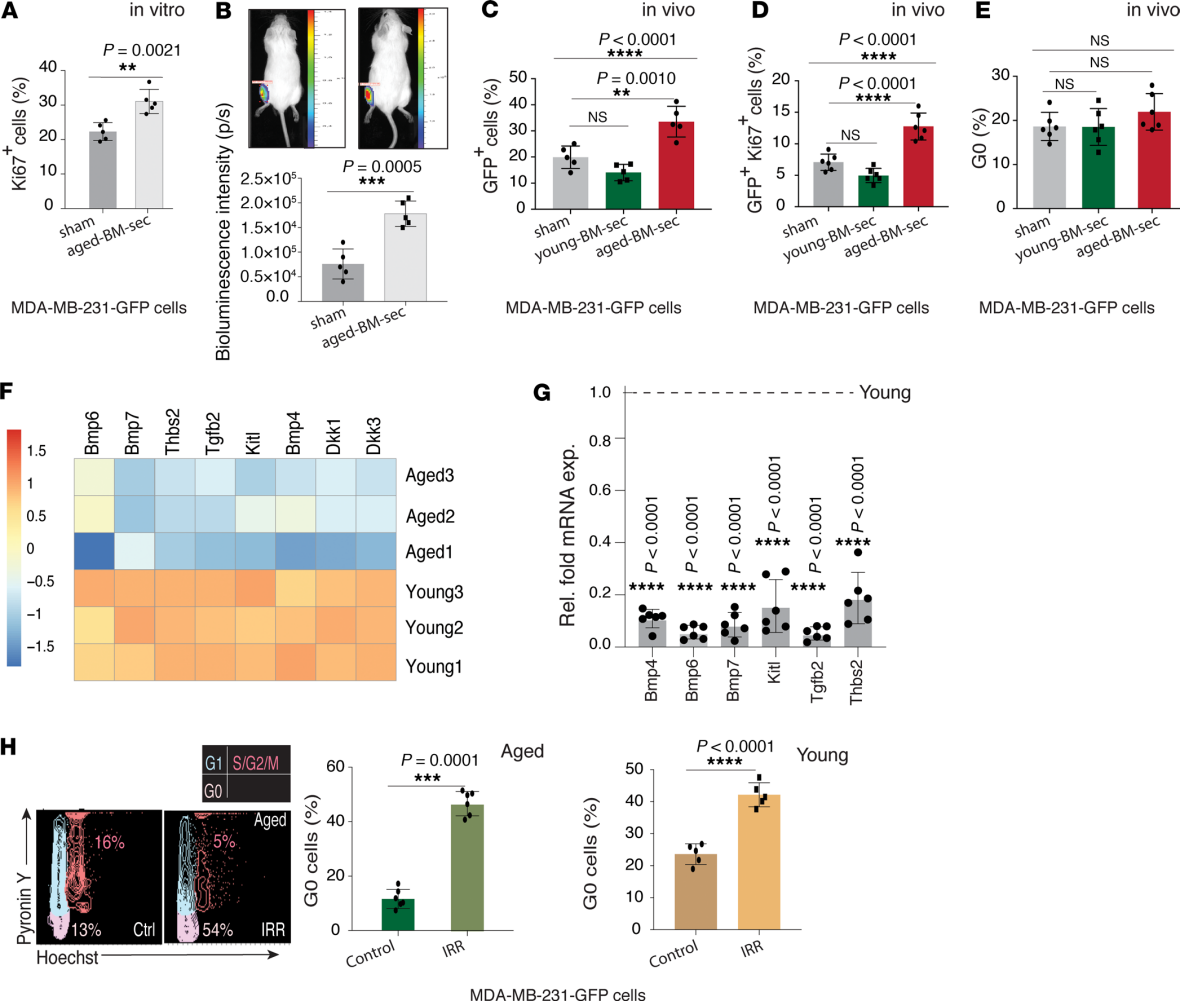
Quiescence-promoting secreted factors decline in the aged BM microenvironment. (**A**) FACS quantification of proliferating MDA-MB-231-GFP cells in culture by Ki-67 immunostaining in PBS and aged BM secretome–treated (aged-BM-sec) cells. Data represent mean ± SD (*n* = 5 replicates); 2-tailed unpaired *t* test. (**B**) Bioluminescence analysis of sham (PBS) and aged-BM-sec injected in young mice tibiae along with cancer cells. Data represent mean ± SD (*n* = 5 replicates); 2-tailed unpaired *t* test. (**C**) FACS quantification of the frequency of MDA-MB-231-GFP cells in single-cell suspensions of tibiae from sham- (PBS-), young-BM-sec– (secretome from young 5-week-old mice), and aged-BM-sec–injected (secretome from aged 70-week-old mice) groups. Data represent mean ± SD (*n* = 5 replicates); 1-way ANOVA by Dunnett’s multiple-comparisons test. (**D**) FACS quantification of Ki-67^+^ MDA-MB-231-GFP cells in sham- (PBS-), young-BM-sec–, and aged-BM-sec–injected tibiae from young mice. Data represent mean ± SD (*n* = 6 replicates); 1-way ANOVA by Dunnett’s multiple-comparisons test. (**E**) FACS quantifications of the quiescent (G_0_) MDA-MB-231-GFP cells in young tibia, based on Hoechst and Pyronin Y staining of single-cell suspensions of the tibiae. Data represent mean ± SD (*n* = 6 replicates); 1-way ANOVA by Dunnett’s multiple-comparisons test. (**F**) The heatmap shows the most significantly differentially regulated secreted factors involved in maintaining stem and cancer cell quiescence. Color intensity indicates row-scaled-normalized log_2_(cpm) expression values. (**G**) qPCR analysis of *Bmp4*, *Bmp6*, *Bmp7*, *Kitl*, *Tgfb2*, and *Thbs2* expression (normalized to *Actb*) by aged tibiae relative to young (Rel. fold mRNA exp.). Data represent mean ± SD (*n* = 6 replicates); 2-tailed unpaired *t* tests. (**H**) Representative FACS contour plots show Hoechst and Pyronin Y staining on gated GFP^+^ MDA-MB-231 cells in single-cell suspensions of radiation-treated and control aged mouse tibiae that were injected with MDA-MB-231-GFP cells 2 weeks after intratibial injections. The left graph shows quantification of quiescent MDA-MB-231-GFP cells in radiation-treated (IRR) aged mice. Data represent mean ± SD (*n* = 6 replicates); 2-tailed unpaired *t* tests. The right graph shows quantification of quiescent MDA-MB-231-GFP cells in radiation-treated young mice. Data represent mean ± SD (*n* = 5 replicates); 2-tailed unpaired *t* tests. **P < 0.01, ***P < 0.001, ****P < 0.0001.

To delineate the molecular mediators of these age-dependent changes, we performed RNA-Seq of young and aged murine bones. The correlation analysis of RNA-Seq samples showed a low variation within each sample group, demonstrating distinct profiles of individual sets of young and aged murine bones (Figure 3A). Differential gene expression analysis with an FDR-adjusted *P* value less than 0.01 and an absolute log_2_ fold change ±1 identified 7280 genes differentially expressed between young and aged bones (Figure 3B). Further, generally applicable gene-set enrichment for pathway analysis (GAGE) was performed to unravel the biological relevance of the RNA-Seq data set. Collectively, GAGE analysis displayed a significant upregulation of 98 (*q* < 0.000001) gene sets in the aged bones. The top upregulated gene sets showed a striking enrichment of cell cycle and proliferation components, and immune system processes in aged bones (Figure 3, A and C). Further analyses revealed that several cytokines with known roles in promoting cell proliferation, such as interleukins Il1b, Il6, Il27, and Il1f9; CCl4 and Ccl5; TNF superfamily member 14 (Tnfsf14); and lymphotoxin β (Ltb) were upregulated in aged compared with young bones (Figure 3D). Quantitative PCR (qPCR) analysis of the most differentially regulated cytokines, such as Il1b and Il6, confirmed and validated the RNA-Seq results (Supplemental Figure 1E). Next, we investigated the role of these age-dependent growth factors and cytokines in regulating the fate of DTCs in bones. To investigate the involvement of BM-derived secretory factors, the acellular BM supernatant (secretome) from aged mice was isolated from tibial bones by the centrifugation method. MDA-MB-231 and MCF-7 cells in culture were then exposed to secretome derived from the bones of aged mice. This treatment with secretome from aged mice resulted in a significant increase in expansion of MCF-7 and MDA-MB-231 cells as compared with control PBS-treated cells (Figure 4A and Supplemental Figure 2A). To further assess the in vivo function of the aged BM secretome on cancer cell proliferation during bone metastasis, MDA-MB-231 cells were intratibially injected into young mice with or without aged BM secretome. Quantification of cancer cells by flow cytometry (based on their GFP expression) and bioluminescence in transplanted mice suggested that the treatment with secretome from the aged mice led to the rapid expansion of MDA-MB-231 and MCF-7 cells in bones (Figure 4, B and C, and Supplemental Figure 2B). Further, there was a significant increase in proliferating cells, as determined by the quantifications of Ki-67^+^ cancer cells (Figure 4D and Supplemental Figure 2C). However, quantifications of the quiescent cancer cell fraction by Hoechst and Pyronin Y staining showed no obvious difference between the aged bone secretome– and PBS-treated young mice (Figure 4E). Remarkably, several factors involved in maintaining stem and cancer cell quiescence such as bone morphogenetic protein 4 (Bmp4), Bmp6, and Bmp7; kit ligand (Kitl); Tgfb2; Dickkopf-related protein 1 (Dkk1) and Dkk3; and thombospondin2 (Thbs2) were upregulated in bones of young mice (Figure 4, F and G, and Supplemental Figure 6B).

### Radiation and chemotherapy induce a quiescence-promoting BM microenvironment.

**Figure 5:**
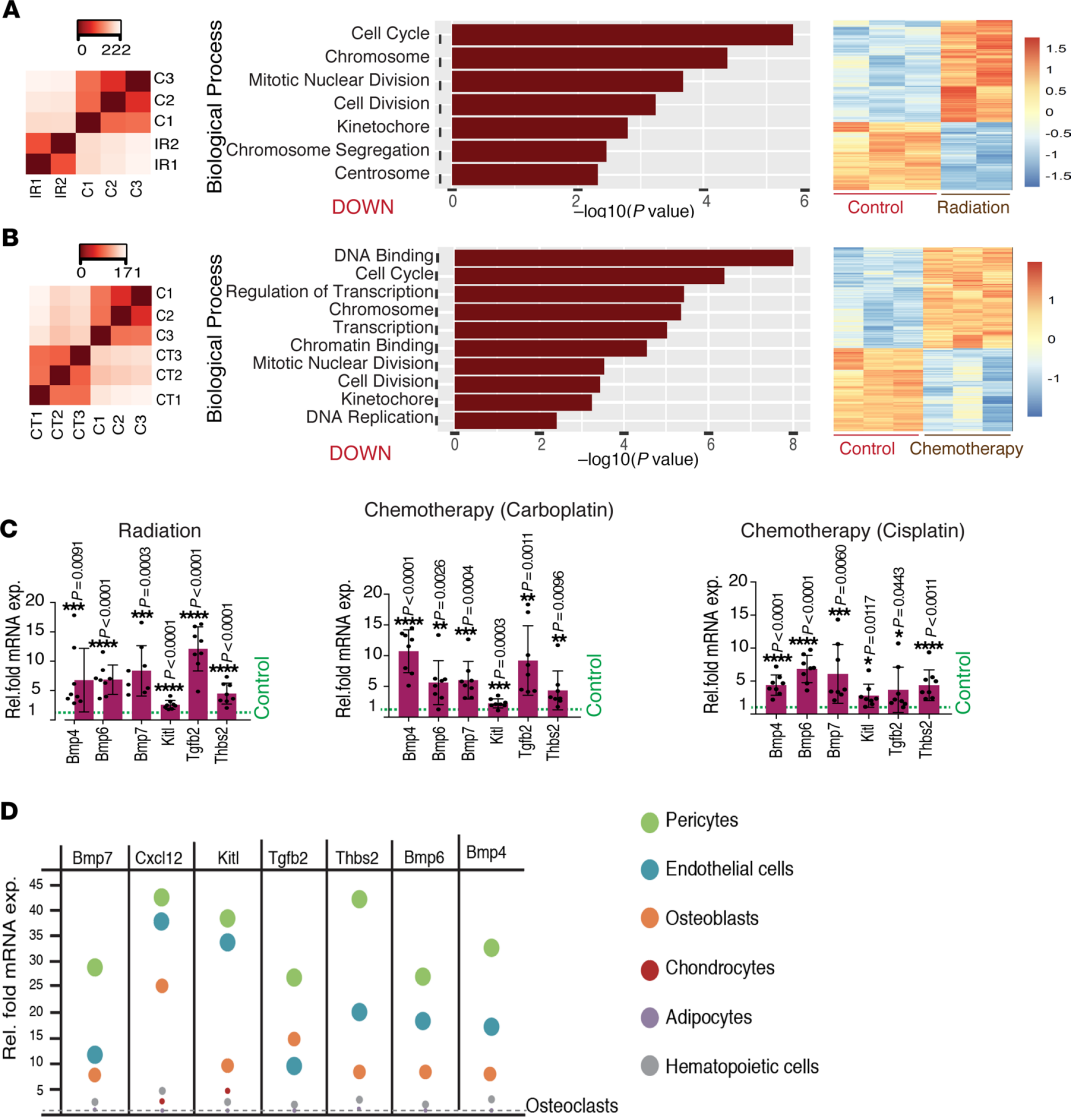
Radiation and chemotherapy induce upregulation of quiescence-promoting factors in the bones via pericytes. (**A** and **B**) The left heatmap shows data quality assessment by hierarchical sample-to-sample distance clustering. Variance-stabilizing transformation of the RNA-Seq read count for all the samples was used to calculate the sample-to-sample Euclidean distance (color scale) for hierarchical clustering. Controls (C1–C3), radiated (IR1–IR2), or chemotherapy-treated (CT1–CT3) whole tibial bones from young mice. GSEA of the radiated bones in comparison to bones from control mice. The most significant biological processes were assessed by GAGE, with *q* < 0.000001. The most differentially downregulated biological processes are shown in the bar graphs. The *y* axis shows the GO terms, whereas the *x* axis represents the enrichment scores of these terms. The right heatmap in **A** shows 4136 differentially expressed genes between the radiated bones in comparison to bones from the control mice (FDR-adjusted *P* value cutoff <0.01, log_2_ fold change ±1). The right heatmap in **B** show 3119 differentially expressed genes between carboplatin-treated and control bones (FDR-adjusted *P* value cutoff <0.01, log_2_ fold change ±1). Color intensity represents row-scaled-normalized log_2_(cpm) expression, whereas the columns represent the replicate of each samples. (**C**) qPCR analysis of *Bmp4*, *Bmp6*, *Bmp7*, *Kitl*, *Tgfb2*, and *Thbs2* expression (normalized to *Actb*) by radiated or chemotherapy-treated tibiae relative to controls from young mice. Data represent mean ± SD (*n* = 8 replicates), and 2-tailed unpaired *t* tests. (**D**) Bubble graph shows the qPCR analysis of *Bmp7*, *Cxcl12*, *Kitl*, *Tgfb2*, *Thbs2*, *Bmp4*, and *Bmp6* expression (normalized to *Actb*) by different cell types (as indicated in the figure) in BM from young mice in comparison to levels in osteoclasts. Data represent mean (*n* = 5 replicates). *P < 0.05, **P < 0.01, ***P < 0.001, ****P < 0.0001.

Downregulation of quiescence-promoting factors in the aged BM microenvironment suggests that cancer cells in aged mouse bones will be highly susceptible to radiation and chemotherapy due to loss of quiescence. In contrast, exposure to radiation in young and aged mice resulted in a dramatic increase in the quiescent cancer cell fraction (Figure 4H). This led us to investigate the radiation-altered BM microenvironment that promoted quiescence of cancer cells. We performed RNA-Seq to analyze gene expression changes in the bones of mice treated with radiation or chemotherapy (Figure 5, A and B, and Supplemental Figure 3A). GAGE analysis of the RNA-Seq data showed that cell cycle and proliferation processes were downregulated after an exposure to radiation or chemotherapy (Figure 5, A and B). Interestingly, the quiescence-promoting factors — *Bmp4*, *Bmp6*, *Bmp7*, *Kitl*, *Tgfb2*, *Thbs2*, *Dkk1*, and *Dkk3* — that were found to be downregulated in the aged bones were upregulated upon an exposure to radiation and chemotherapy (Supplemental Figure 3, B and C). In addition, *Cxcl12*, a stromal cell–derived α chemokine member known to be an important regulator of quiescence in stem and cancer cells, was upregulated after radiation and chemotherapy (Supplemental Figure 3, B and C). qPCR and ELISA analysis confirmed that these factors were upregulated in normal bones and bones with metastatic cancer cells after radiation or chemotherapy in young and aged mice (Figure 5C and Supplemental Figure 3, D–L). While quiescence-promoting secreted factors were significantly increased, *Il1b* and *Il6* were downregulated under these conditions (Supplemental Figure 4, A–E). The above findings established that both radiation and chemotherapy treatment promoted release of quiescence-inducing factors in the BM microenvironment.

### Radiation and chemotherapy induce bone-specific pericyte expansion.

**Figure 6:**
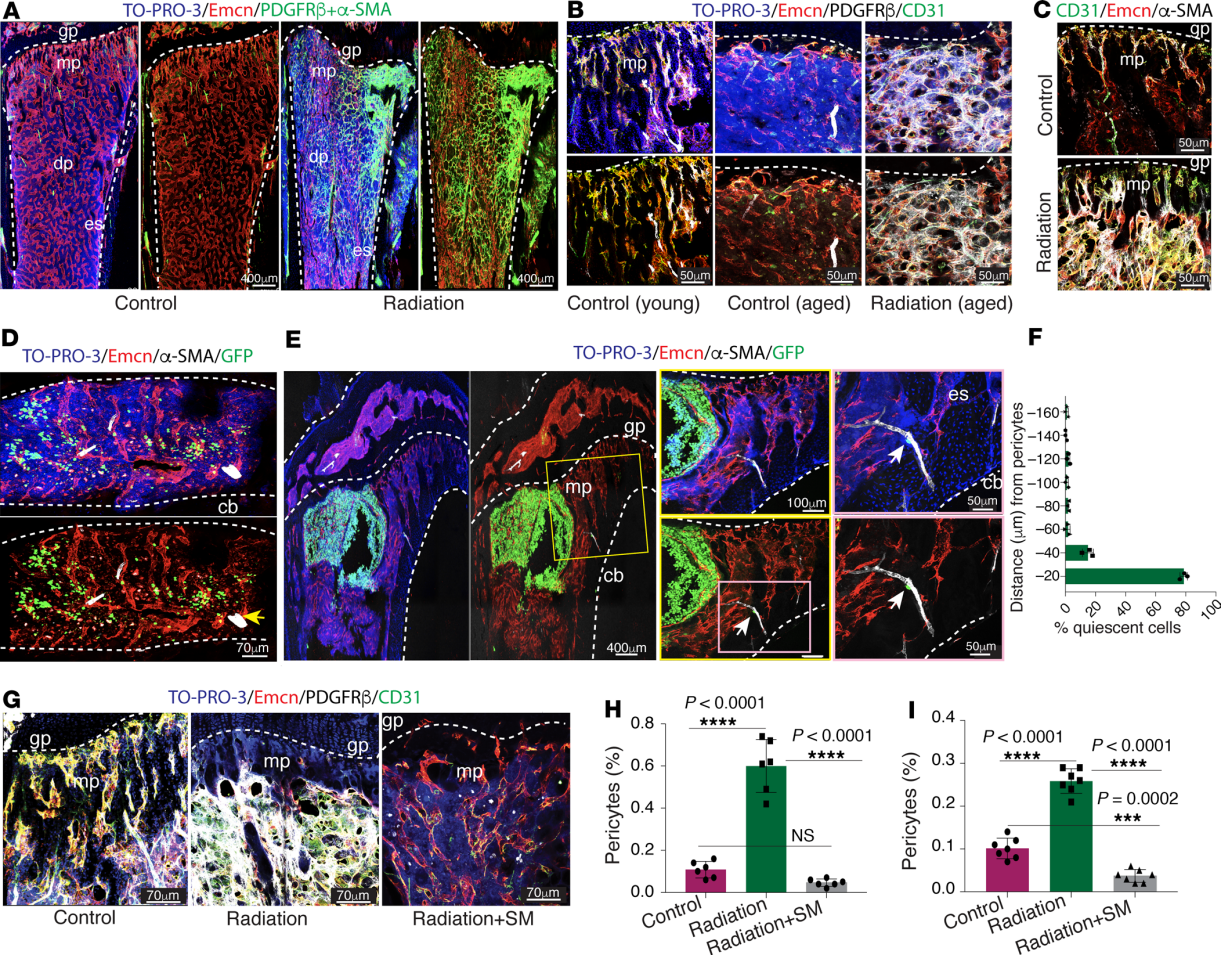
Radiation and chemotherapy drive bone-specific expansion of the pericytes. (**A**) Representative tile scan confocal images show PDGFRβ + α-SMA (green) and endomucin (Emcn; red) immunostaining on thick sections (150 μm) of young tibia exposed to radiotherapy or control mouse tibia; nuclei, TO-PRO-3 (blue). The dotted lines mark the growth plate (gp) or endosteum (es). mp, metaphysis; dp, diaphysis. Scale bars: 400 μm. (**B**) Confocal images with PDGFRβ (white), CD31 (green), and endomucin (red) immunostaining on thick sections from young tibia and from aged tibia either control or exposed to radiation. Scale bars: 50 μm. (**C**) Representative confocal 3D images with α-SMA (white), CD31 (green), and endomucin (red) immunostaining on sections of young tibia from control and irradiated mice, i.e., metaphysis or growth plate. Scale bars: 50 μm. (**D**) Representative 3D confocal images with α-SMA (white) and endomucin (red) immunostaining on a young tibia with bone metastasis 19 days after intracardiac injection of MDA-MB-231-GFP cells. cb, compact bone. Scale bars: 70 μm. (**E**) Tile scan (left panel) confocal images show α-SMA (white) and endomucin (red) immunostaining in a young, 10-week-old tibia with bone metastasis 3-weeks after intracardiac injection of MDA-MB-231-GFP cells. Arrows highlight the isolated single MDA-MB-231-GFP cells attached to an α-SMA artery. Scale bars: 400 μm (left), 100 μm (center), 50 μm (right). (**F**) Distribution of quiescent MDA-MB-231-GFP cells in tibial bone sections from young mice. Seventy-nine dormant cells were analyzed and quantified from 3 mice. (**G**) Confocal images show PDGFRβ (white), endomucin (red), and CD31 (green) immunostaining on young, 7-week-old tibiae from control, radiation-treated, and radiation + sunitinib malate–treated (SM) groups. The dotted lines mark the growth plate. Scale bars: 70 μm. (**H**) FACS quantification of pericytes (CD31^–^CD45^–^Ter119^–^PDGFRβ^+^) in a single-cell suspension of long bones from radiation-treated, radiation + sunitinib malate–treated, and control young mice. Data represent mean ± SD (*n* = 6 replicates); 1-way ANOVA with Tukey’s multiple-comparisons test. (**I**) FACS quantification of pericytes (CD31^–^CD45^–^Ter119^–^PDGFRβ^+^) in a single-cell suspension of tibiae from carboplatin-treated, carboplatin + sunitinib malate–treated, and control mice. Data represent mean ± SD (*n* = 7 replicates); 1-way ANOVA with Tukey’s multiple-comparisons test. Representative images were derived from 3 independent experiments. ***P < 0.001, ****P < 0.0001.

To determine the cell type predominantly contributing to the release of quiescence-promoting factors in the BM, we investigated the cellular composition of these microenvironments. Recent studies suggest that skeletal aging is marked by significant changes in the cellular composition of the BM microenvironment (6, 7). Cell surface marker expression analysis corroborated the previously reported changes in cell types in the BM with aging (Supplemental Figure 5, A and B) (9). We purified individual cell types from the BM microenvironment and analyzed the cell-specific expression pattern of quiescence-inducing secretory factors. Specifically, expression analysis of *Bmp4*, *Bmp6*, *Bmp7*, *Kitl*, *Tgfb2*, *Thbs2*, and *Cxcl12* was performed in pericytes, endothelial cells (ECs), osteoclasts, osteocytes, adipocytes, chondrocytes, and hematopoietic cells. Significant upregulation of quiescence-inducing factors was observed in pericytes and ECs in comparison to other cell types in the BM (Figure 5D). Analyses of young and aged bone microenvironments after radiation and chemotherapy (carboplatin treatment) showed a dramatic expansion of pericytes along with blood vessel modifications (Figure 6, A and B, and Supplemental Figure 6A). These PDGFRβ^+^ cells were also positive for α–smooth muscle actin (α-SMA), which is expressed by pericytes (Figure 6, A and C). It should be noted that metastatic cancer cells alone without exposure to radiation did not stimulate the expansion of pericytes in bones (Figure 6D). In line with the above results, single isolated cancer cells were frequently detected in close proximity to pericytes (Figure 6, E and F), suggesting the nonproliferative nature of the microenvironment. Remarkably, PDGFRβ immunostaining of thick sections from heart, kidney, liver, and spleen of these radiated mice showed no difference compared with control mice, indicating that this expansion of pericytes in response to radiation is a bone-specific phenomenon (Supplemental Figure 7A). In contrast to radiation and chemotherapy, aging resulted in a reduction in pericytes (Figure 6B and Supplemental Figure 7B) that led to a decline in cellular quiescence-inducing factors, and thereby loss of cancer cells and HSC quiescence in the aged BM microenvironment. Remarkably, an age-dependent decline in quiescence-inducing factors such as *Bmp4*, *Bmp6*, *Bmp7*, *Kitl*, *Tgfb2*, and *Thbs2* was also observed specifically in bones (Supplemental Figure 8).

It is evident from earlier studies that the dissemination of tumor cells is an early event of metastasis, often occurring before the detection of primary tumors (25, 26). The above results illustrating bone-specific expansion of pericytes and upregulation of quiescence-inducing factors in response to radiation or chemotherapy suggest that DTCs in bones could gain an advantage in escaping conventional therapeutic regimens. This provides a plausible explanation for bone as one of the most frequent sites of metastatic relapse. The age-dependent loss of pericytes (Figure 6B) and quiescence-promoting niches in bone provide signals for reactivation of quiescent DTCs, describing the long latency periods observed before metastatic relapse in bone. Taken together, these findings establish a key role played by bone pericytes in promoting quiescence in response to radiation or chemotherapy.

### Bone-specific expansion of pericytes is driven by type H endothelium–induced PDGF-B signaling.

**Figure 7:**
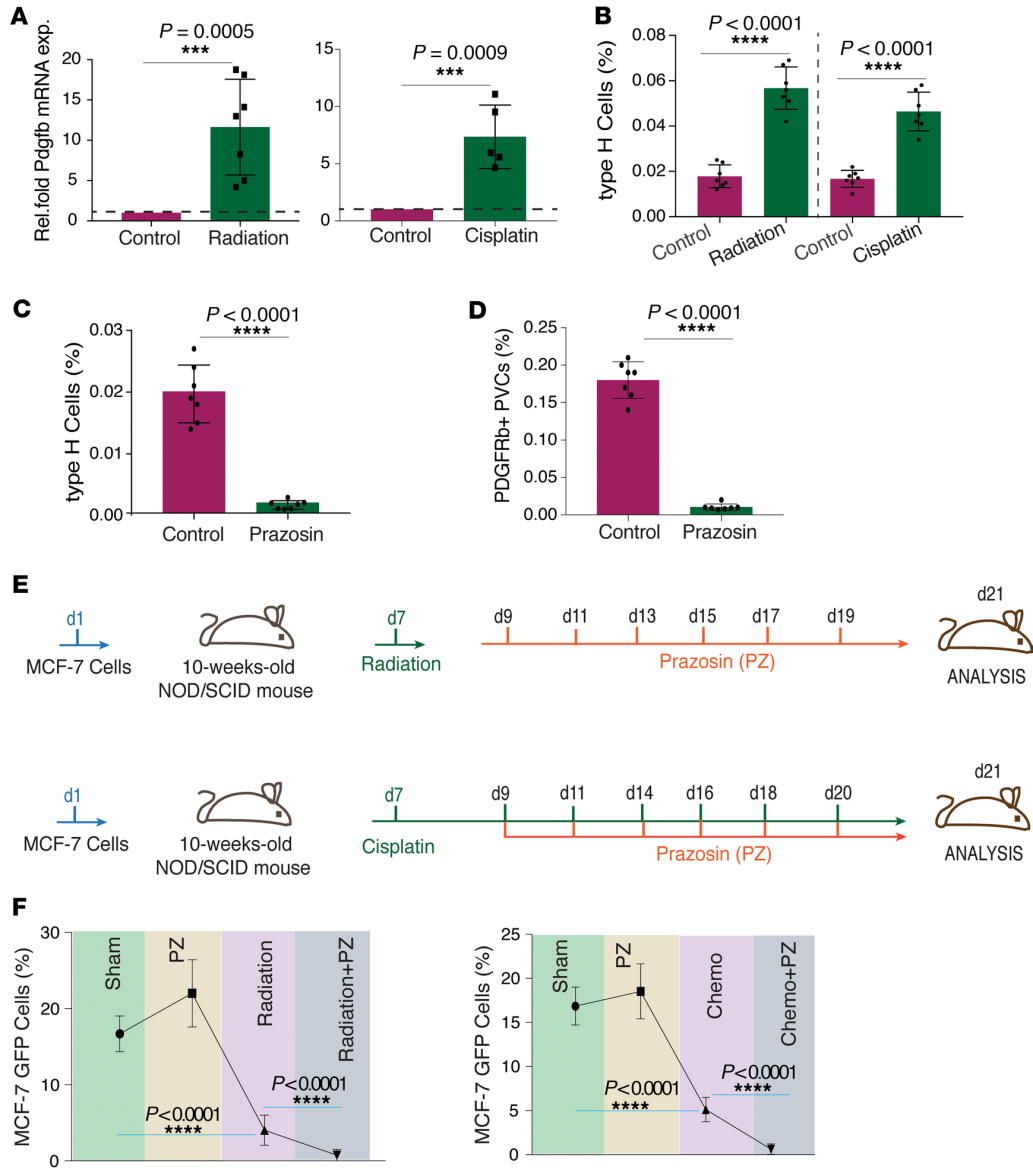
Manipulating blood flow renders bone metastatic cells susceptible to radiation and chemotherapy. (**A**) The left graph shows qPCR analysis of *Pdgfb* expression (normalized to *Actb*) by radiation-exposed relative to control tibiae from young mice. Data represent mean ± SD (*n* = 7 replicates); 2-tailed unpaired *t* test. The right graph shows qPCR analysis of *Pdgfb* expression (normalized to *Actb*) (Rel.fold Pdgfb mRNA exp.) by cisplatin-treated tibiae relative to control tibiae from young mice. Data represent mean ± SD (*n* = 5 replicates); 2-tailed unpaired *t* test. (**B**) FACS quantification of CD31^hi^/endomucin^hi^ type H ECs in irradiated and chemotherapy/cisplatin-treated relative to control tibiae derived from young mice. Data represent mean ± SD (*n* = 7 replicates); 2-tailed unpaired *t* test. (**C**) FACS quantification of CD31^hi^/endomucin^hi^ ECs in prazosin-treated relative to control tibiae from young mice. Data represent mean ± SD (*n* = 7 replicates); 2-tailed unpaired *t* test. (**D**) FACS quantification of PDGFRβ^+^ (CD31^–^CD45^–^Ter119^–^) perivascular cells (PVCs) in prazosin-treated relative to control tibiae from young mice. Data represent mean ± SD (*n* = 7 replicates); 2-tailed unpaired *t* test. (**E**) Schematic representation of the radiation and chemotherapy/cisplatin treatment scheme along with blood flow manipulation/prazosin injections. (**F**) Left panel: FACS quantification of MCF-7 cells based on GFP expression in single-cell suspensions of tibiae from prazosin-treated (PZ), radiation-treated, radiation + prazosin–cotreated, and control mice. Data represent mean ± SD (*n* = 11 replicates); 1-way ANOVA with Dunnett’s multiple comparisons test. Right panel: FACS quantification of MCF-7 cells based on GFP expression in single-cell suspensions of tibiae from prazosin-treated, cisplatin-treated (chemo), chemo + prazosin–cotreated, and control mice. Data represent mean ± SD (*n*=11 replicates); 1-way ANOVA with Dunnett’s multiple-comparisons test. ***P <0.001, ****P <0.0001.

To unravel the mechanism behind bone-specific expansion of PDGFRβ^+^ pericytes, we analyzed expression of the growth factor PDGF-B. Analysis of *Pdgfb* transcripts in bones from mice treated with radiation and chemotherapy (cisplatin) showed upregulation of *Pdgfb* expression compared with control mouse bones (Figure 7A). Analysis of PDGF-BB by ELISA on bone supernatants from young and aged mice treated with radiation and chemotherapy also confirmed the significant increase in PDGF-BB (Supplemental Figure 9A). However, *Pdgfb* expression showed no significant difference in liver, lung, kidney, heart, or brain after radiation (Supplemental Figure 9B). ECs secrete PDGF-B, which binds to PDGFRβ located on the pericyte plasma membrane, activating signal transduction pathways that ultimately promote cell proliferation (39). ECs in bone are heterogenous and composed of different subsets. Further, a specific subset of ECs termed type H (CD31^hi^Emcn^hi^) is known to manifest high expression of PDGF-B. Therefore, we quantified different subsets of ECs in bone after treatment with radiation and chemotherapy. Quantification of these CD31^hi^Emcn^hi^ ECs in these bones demonstrated a significant increase (Figure 7B). In addition, qPCR analysis of *Pdgfb* transcript on purified CD31^hi^Emcn^hi^, CD31^lo^Emcn^lo^, and arterial ECs from bones of radiation- and cisplatin-treated mice confirmed the high levels of *Pdgfb* in type H ECs (Supplemental Figure 9C). This increase in type H endothelium and PDGF-B levels led us to investigate the involvement of PDGFRβ (the receptor for PDGF-B) in order to understand its involvement in mediating the response. Expression of PDGFRβ is crucial for proliferation and expansion of pericytes in response to the growth factor PDGF-B. Sunitinib malate is an indolinone-based tyrosine kinase known to block tyrosine kinase activity of the PDGFRβ and thereby inhibit the proliferation of PDGFRβ^+^ cells. The treatment with sunitinib malate alongside radiotherapy and chemotherapy inhibited the expansion of the PDGFRβ^+^ cells in the mouse BM microenvironment (Figure 6, G–I). Thus, the above findings illustrated that the observed bone-specific expansion of pericytes is mediated by PDGF-B/PDGFRβ signaling.

### Modulation of blood flow enhances the efficacy of therapy by targeting pericytes.

Further, we interrogated the role of blood flow in metastasis, as previously we found that blood flow is a positive regulator of type H ECs in bone (Figure 7C). Here, we confirmed whether blood flow–mediated reduction in type H ECs is associated with a decrease in PDGFRβ^+^ pericytes by treatment with prazosin, a pharmacological inhibitor of α_1_-adrenergic receptor (40) that reduces blood flow to bone (Figure 7D). Similar phenotypic changes in blood vessels and pericytes were observed in mice treated with another flow-reducing drug, clonidine (Supplemental Figure 9, D and E). These data support the potential relationship among age-dependent reduction in blood flow to bone (41), reduction in type H ECs and pericytes and thus decline in quiescence-promoting factors, and development of macrometastasis. Further, these data also indicate that pericyte expansion in bone can be manipulated by alterations in blood flow; specifically, reducing blood flow provides the opportunity to inhibit pericyte expansion.

Based on the above findings, we tested whether modulation of blood flow along with chemotherapy and radiation provides a therapeutic approach to inhibit quiescent DTCs by reducing pericyte expansion in bone. Toward this, we treated mice with prazosin in combination with radiation or chemotherapy after intracardiac injection of MCF-7 cells. After the radiation treatment, we found that a subset of metastasized cancer cells escaped and survived the treatment in the BM; we quantified these cells based on their GFP expression by flow cytometry. Remarkably, prazosin administration in combination with radiation led to a significant reduction in radiation-resistant cells (Figure 7, E and F), suggesting increased susceptibility of cancer cells in bones to radiation treatment. Similarly, administration of prazosin along with cisplatin led to a substantial reduction in chemoresistant cells (Figure 7, E and F). Thus, the reduction of blood flow in radiation and chemotherapy treatments targeted resistant cancer cells by preventing cancer cells from acquiring a quiescent phenotype by modulating the bone microenvironment. These findings suggest that inhibiting radiation- and chemotherapy-induced expansion of pericytes by modulation of blood flow has the potential to improve current paradigms of anticancer treatments to manage or prevent bone metastasis (Supplemental Figure 10).

## Discussion

In this study, we found that age-dependent changes in cell-extrinsic factors, particularly secretory factors, promote DTC proliferation while inhibiting the quiescence of stem and cancer cells in bone. Age-dependent cellular changes in the vascular compartment of the BM microenvironment contribute to these changes in secretory factors. Emerging evidence suggests the importance of EC- and pericyte-derived signals and secretory factors (also called angiocrine factors) in development, homeostasis, repair, and regeneration (6, 39, 42–44). Aging in ECs is associated with diminished release of PDGF-B, which in turn leads to decreased PDGFRβ^+^ perivascular cells in the microenvironment. Both endothelial and perivascular cells were identified as contributing to the majority of angiocrine factors that regulate stem and cancer cell status in bone (35, 45, 46). Specifically, type H capillaries seem to play a central role, as they express high levels of PDGF-B compared with type L capillaries and are associated with PDGFRβ^+^ perivascular cells. Type H capillaries were earlier identified as supporting long-term HSCs by providing arteriolar niches, which decrease with age (8, 33). In addition to HSCs, the status of MSCs and DTCs is also known to change with their interplay with the microenvironment (10, 14, 25). We find that similar to HSCs, DTCs in arteriolar niches are generally single cells, indicating the quiescent nature of this microenvironment. It also indicates the importance of external signals in maintaining the status of stem cells, though further understanding is needed. Since this microenvironment also affects MSCs, imaging of specific microenvironments supporting MSCs will be beneficial in our understanding of MSCs and their lineage. Studies suggesting a perivascular origin of MSCs support this quiescence-promoting microenvironment in bone. As the BM microenvironment is a key regulator of local stem and cancer cell proliferation and quiescence dynamics, it is likely to trigger reactivation of dormant DTCs upon aging. This also suggests that future therapeutic interventions should target the promiscuous interactions of cancer cells with the surrounding microenvironment rather than DTCs.

Our study also reveals that both radiation and chemotherapy have the intrinsic potential to remodel the BM microenvironment via expansion of vascular niches, in particularly pericytes. Earlier studies have illustrated involvement of pericytes in tumor angiogenesis (43, 44). However, their role in and response to radiation and chemotherapy remain unknown. This remodeling of vascular niches mediates the reprogramming of the BM microenvironment through the quiescence-promoting secretome of pericytes and ECs, and thereby contributes toward chemoresistance. Thus, our results suggest that to avoid the development of chemoresistance and age-dependent metastatic relapse, novel therapeutic interventions might target not DTCs, but rather the BM microenvironment, in particular quiescence-promoting vascular niches or the ability of DTCs to communicate with surrounding microenvironments. Further investigations will provide an understanding of whether individual factor(s) or a combination of factors are required to maintain these niches. Targeting blood flow along with the therapeutic regimens is an efficient approach to target endothelium and perivascular cells to promote expansion of DTCs in bone. With the recent identification of type H blood vessels in the human skeletal system, we believe targeting ECs and perivascular cells by blood flow will be beneficial in clinical settings.

## Methods

### Genetically modified and aged mice.

C57BL/6J, mT/mG (catalog 026862), and NOD/SCID mice were purchased from the Jackson Laboratory. C57BL/6J mice were used for WT bone analysis, unless stated otherwise. For tumor cell injection experiments, mice at ages 4–6 weeks and 55–65 weeks were chosen for young and aged group sets, respectively. For all other experiments, young mice ranged between 4 and 10 weeks and aged mice were between 55 and 70 weeks. Before the experiments began, mice were randomly divided into cages. Cages were subsequently randomly allocated for treatment. For experimental metastasis assays, MDA-MB-231 (Caliper Life Sciences), MCF luciferase GFP (GeneCopoeia), and ZR-75-1 GFP (GenTarget Inc.) cells were injected into the left cardiac ventricle of NOD/SCID mice with a 26½ gauge needle. Successful injection was confirmed by pumping arterial blood into the syringe. For intratibial injections, 2.5 × 10^5^ of the above-mentioned breast cancer cells per 10 μL PBS were injected into left tibia, and 10 μL PBS was injected into right tibia of the same mouse. Mice were dissected at 15, 19, 21, or 28 days after intracardiac or intratibial injections.

### Radiation, chemotherapy, and prazosin treatment.

When indicated, adult C57BL/6J WT mice were whole-body irradiated with a single dose of 900 rad (Gammacell irradiator) and euthanized 5 days later. For chemotherapy, adult C57BL/6J mice were treated with carboplatin or cisplatin as indicated. Both carboplatin and cisplatin were prepared fresh in saline. Carboplatin was injected intraperitoneally, 30 mg/kg/mouse 3 times each week for 6 doses. Cisplatin was injected intraperitoneally, 10 mg/kg/mouse 3 times each week for 3 doses. For prazosin treatment, mice were given 0.5 mg kg^−1^ body weight dose every alternate day for 6 days.

### Cell culture and reagents.

MDA-MB-231, ZR-75-1, MCF-7, and ZR-75-1 GFP cell lines were maintained on tissue culture plastic in DMEM growth medium supplemented with 10% (vol/vol) FBS, 1% (vol/vol) penicillin-streptomycin, and 1% (vol/vol) nonessential amino acid solution in a humidified incubator at 37°C with 5% carbon dioxide. Subconfluent cultures (80% confluence) were briefly treated with trypsin. Cells were harvested and resuspended in 10% FBS/DMEM and then washed twice in 0.1% FBS/DMEM. They were plated at 5 × 10^5^ cells/well in 6-well plates and incubated with 0.1% FBS/DMEM for 6 hours. After 6 hours, 50 μL aged BM secretome/supernatant was added, and cells were cultured for 24 hours. In control wells, 50 μL PBS was added, and cells were cultured for 24 hours. After 24 hours, cells were harvested and used immediately for Ki-67 immunostaining. PKH26 labeling was performed as described earlier (47).

### Bone immunohistochemistry.

Freshly dissected bones collected from WT mice or mutants and their control littermates were immediately fixed in ice-cold 4% paraformaldehyde solution for 4 hours. Decalcification was carried out with 0.5 M EDTA at 4°C with constant shaking. Decalcified bones were immersed into 20% sucrose and 2% polyvinylpyrrolidone (PVP) solution for 24 hours. Finally, the tissues were embedded and frozen in 8% gelatin (porcine) in the presence of 20% sucrose and 2% PVP. For immunofluorescence staining and morphological analyses, sections were generated using low-profile blades on a Leica CM3050 cryostat.

For immunostaining, bone sections were air dried, permeabilized for 10 minutes in 0.3% Triton X-100, blocked in 5% donkey serum at room temperature (RT) for 30 minutes, and probed with primary antibodies diluted in 5% donkey serum in PBS for 2 hours at RT or overnight at 4°C. All primary antibodies are listed in Supplemental Table 1.

After primary antibody incubation, sections were washed with PBS 3 times and incubated with appropriate Alexa Fluor–coupled secondary antibodies (1:400, Molecular Probes) for 1 hour at RT. The following secondary antibodies were used: donkey anti-Rat IgG (H+L) Alexa Fluor 594 (A21209, Thermo Fisher Scientific), donkey anti-goat IgG (H+L) Alexa Fluor 488 (A11055, Thermo Fisher Scientific), donkey anti-goat IgG (H+L) Alexa Fluor 647 (A21447, Thermo Fisher Scientific), and donkey anti-rabbit IgG (H+L) Alexa Fluor 488 (A21206, Thermo Fisher Scientific).

Nuclei were counterstained with TO-PRO-3. Sections were thoroughly washed with PBS before being mounted using Fluoromount-G (SouthernBiotech). Finally, coverslips were sealed with nail polish.

### Image acquisition and quantitative analysis.

Immunofluorescence stainings were analyzed at high resolution with a Zeiss laser scanning confocal microscope, LSM-880. *Z*-stacks of images were processed and 3D reconstructed with Imaris software (version 9, Bitplane). Imaris, Photoshop, and Illustrator (Adobe) were used for image processing. All quantifications was done with ImageJ (NIH) and Imaris software on high-resolution confocal raw data.

### qPCR.

Single-cell suspensions from bones were used for analyses of expression levels of mRNA. Samples were lysed in the lysis buffer of the RNeasy Mini Kit (QIAGEN). Total RNA was isolated according to the manufacturer’s protocol. A total of 100 ng RNA per reaction was used to generate cDNA with the iScript cDNA Synthesis System (Bio-Rad). qPCR was performed using PowerUp SYBR Green Real-Time PCR Master Mix (Thermo Fisher Scientific) with customized primer pairs. Sequences of primer pairs used in the study are listed in Supplemental Table 2. For analysis of mRNA expression levels from whole bones, dissected femurs or tibiae were immediately crushed finely, digested with collagenase, and centrifuged to obtain a pellet, which was then lysed into lysis buffer of the RNeasy Mini Kit (QIAGEN). For cells in culture, culture medium was completely removed, and cells were immediately lysed with lysis buffer. A total of 500 ng RNA per reaction was used to generate cDNA with the iScript cDNA Synthesis System (Bio-Rad), and further processed as described above.

### RNA isolation and library preparation for RNA sequencing.

For RNA sequencing of the analysis of mRNA, expression levels in a purified cell population from single-cell-suspension cells were lysed in lysis buffer of the RNeasy Mini Kit (QIAGEN). Total RNA was isolated according to the manufacturer’s protocol. RNA quality was checked using a 2100 BioAnalyzer (Agilent). TruSeq Stranded RNA Library Prep Kit (Illumina) was used according to the manufacturer’s instructions for the preparation of sequencing libraries. Sequencing was carried out at the LMS MRC Genomics Facility, Imperial College London, on an Illumina NextSeq 500 platform with 100-nt-length paired end. RNA-Seq data was uploaded to the Array Express database (https://www.ebi.ac.uk/arrayexpress/) with accession number E-MTAB-6872.

### Quality assessment.

Data quality assessment of raw sequence data of mouse bone were analyzed by FastQC (version 0.11.5, http://www.bioinformatics babraham.ac.uk/projects/fastqc/).

### Alignment to reference genome.

Raw reads were mapped to genome assembly (GRCm38), using TopHat-2 with setting the parameter (version 2.0.13; [tophat2-g 2]) (48). Mouse genome as well as Bowtie index were downloaded from the iGenome portal (https://support.illumina.com/sequencing/ sequencing_software/igenome.html). Quantification of the aligned reads on a per-gene basis was obtained using HTSeq with the following settings (version 0.9.1; [htseq-count-mode = intersection-nonempty − stranded = reverse]) (49).

### Differential gene expression analysis.

To explore similarities and dissimilarities between samples, count data were normalized using the variance stabilizing transformation (VST) function from the DESeq2 package (50). Differential gene expression analysis between the aged versus young, irradiation treated versus control, and chemotherapy-treated versus control mouse bones was performed using DESeq2. Differentially regulated genes are listed with FDR-adjusted *P* cutoff <0.01 and an absolute log_2_ fold change ±1. The Ensembl ID was annotated to gene symbols and NCBI Gene using biomaRt (version 3.4.1, BioConductor). The color intensites in the heatmaps indicate the row-scaled-normalized log_2_(cpm) expression values. Differentially regulated genes for different contrasts are listed in Supplemental Tables 3–5.

### Functional analyses of genes.

The overrepresented gene ontology (GO) terms were determined using GAGE (version 3.4.1, BioConductor) (51). The mouse annotation “org.Mm.eg.db” (version 3.4.1, Bioconductor) was used for this functional annotation. Gene set enrichment analysis was performed based on one-on-one comparison between aged and young bone samples, where a young sample was used as a reference sample, whereas an old sample was taken as an expression data set. The analysis steps were followed as described in the vignettes of GAGE. Here, we explored expression changes in one direction (either up- or downregulation) in the gene sets. Significance of enrichment was calculated by 2-sample *t* tests; *P* values were adjusted for multiple testing using the Benjamini-Hochberg method, and *q* < 0.000001 was considered significant. Similarly, GAGE analysis was performed for irradiation-treated versus control and chemotherapy-treated versus control mouse bone. Significant GO terms were selected using FDR-adjusted *P* < 0.001 for irradiation-treated versus control mouse bones and FDR-adjusted *P* < 0.000001 for chemotherapy-treated versus control mouse bones. Enriched GO terms in downregulated gene sets are listed in Supplemental Tables 6–8.

### Isolation of pericytes for qPCR.

Femurs and tibiae were collected, crushed in sterile condition, digested with collagenase A (Sigma-Aldrich) at 37°C for 45 minutes, and passed through a 40-μm filter to obtain single-cell suspensions.

PDGFRβ (AB32570, Abcam) antibodies raised in rabbit were used to isolate pericytes by magnetic bead–based separation. Positive cells were then separated by using anti-rabbit magnetic beads (Dynabeads M-280 Sheep Anti-Rabbit, Thermo Fisher Scientific) following the manufacturer’s instructions. Samples were lysed in the lysis buffer of the RNeasy Mini Kit (QIAGEN). Total RNA was isolated according to the manufacturer’s protocol. cDNA conversion was performed by using a High-Capacity cDNA Reverse Transcription Kit (Applied Biosystems). qPCR was performed using PowerUp SYBR Green Real-Time PCR Master Mix (Thermo Fisher Scientific) with customized primer pairs. Sequences of primer pairs used in the study are listed in Supplemental Table 5.

### Transplantation.

Competitive repopulation assays were performed using mT/mG donor mice with Tomato^+^ cells and WT Tomato^–^ C57BL/6 recipient mice. Equivalent volumes of Tomato^+^ BM cells were collected from mT/mG donor mice and transplanted into lethally irradiated (12 Gy) C57BL/6 recipient mice with 0.3 × 10^6^ competitor WT cells from C57BL/6 mice. mT/mG^+^–mT/mG^–^ chimerism of recipients’ C57BL/6 blood was analyzed up to 4 months after transplantation using flow cytometric analysis.

For MSC transplantations, intratibial injections were performed using the mT/mG donor mice with Tomato^+^ cells in C57BL/6 recipient mice. Femurs and tibiae from 8-week-old mT/mG mice were collected, crushed in sterile condition, digested with collagenase A (Sigma-Aldrich) at 37°C for 45 minutes, and passed through a 40-μm filter to obtain single-cell suspensions. The single-cell suspensions obtained were subjected to lineage cell depletion (130-090-858, Miltenyi Biotec). After lineage cell depletion, PDGFRα^+^ cells were isolated using magnetic bead–based separation using an PDGFR antibody (ab90967, Abcam) raised in rabbit. PDGFRα^+^ cells were separated by using anti-rat magnetic beads (Dynabeads M-280 Sheep Anti-Rat) following the manufacturer’s instructions. Isolated cells were then labeled with PKH67 (PKH67GL-1KT, Sigma-Aldrich). For intratibial injections, 1 × 10^4^ of these isolated cells in 10 μL PBS were injected into the left tibia. Mice were dissected 10 weeks after intratibial injections, and the frequency of Tomato^+^ PDGFRα^+^ cells was assessed by flow cytometry immunostaining with PDGFRα antibody.

### Isolation of BM cell subsets for qPCR.

Femurs and tibiae were collected, crushed in sterile condition, digested with collagenase A (Sigma-Aldrich) at 37°C for 45 minutes, and passed through a 40-μm filter to obtain single-cell suspensions. Different cell fractions were isolated using magnetic bead–based cell separation. Pericytes were isolated using PDGFRβ antibody as described above. For isolation of osteoclasts, anti-RANK antibody was used. To obtain BM adipocytes, osteoblasts, and chondrocytes, MSCs were isolated as described above by magnetic bead–based separation after lineage depletion. These MSCs were then subjected to adipocyte, osteocyte, and chondrocyte differentiation using differentiation media and supplements (R&D Systems). BM ECs were isolated using endomucin antibody. Arterial ECs were isolated using BCAM1 antibody. Type H ECs were isolated by flow cytometry as described previously (9).

### Flow cytometry.

For flow cytometric analysis, tibiae and femurs were collected and cleaned thoroughly to remove adherent muscles. Tibiae and femurs were crushed in ice-cold PBS with mortar and pestle. Whole BM was digested with collagenase incubation at 37°C for 20 minutes. For the analysis of total ECs in bone, tibiae were processed as described above to obtain single-cell suspensions and stained with biotin-coupled CD45 (553077, BD Bioscience) or Ter119 (559971, BD Bioscience) antibodies for 45 minutes. After washing in PBS, cells were stained with streptavidin PE-Cy5 (554062, BD Bioscience) and Alexa Fluor 488–conjugated CD31 (FAB3628G, R&D Systems) antibodies for 45 minutes. After washing, cells were acquired on a FACS instrument (LSRFortessa, BD Bioscience) and analyzed using FACSDiva (version 6.0, BD Bioscience). Total bone ECs were quantified as CD31^+^CD45^–^Ter119^–^.

For analysis of HSC frequency in the BM, BM cells were isolated by crushing long bones with mortar and pestle in Ca^2+^- and Mg^2+^- free PBS supplemented with 2% heat-inactivated bovine serum. Cells were drawn by passing through a 25G needle several times and filtered with a 70-μm filter. The following antibodies were used to stain HSCs: biotin-labeled lineage markers (CD5, CD11b, CD45R, Ly-6G, and Ter119), c-Kit, Sca-1, CD48, and CD150 antibodies (for details, see Supplemental Table 4).

For the enrichment and sorting of hematopoietic stem and progenitor cells, BM cells were isolated by crushing long bones with mortar and pestle in Ca^2+^- and Mg^2+^- free PBS supplemented with 2% heat-inactivated bovine serum. Cells were drawn by passing through a 25G needle several times and filtered with a 70-μm filter. Single-cell suspensions obtained were subjected to lineage depletion (MACS, Miltenyi Biotec). Lineage-depleted BM cells were then stained with c-Kit and Sca-1 antibodies (for details, see Supplemental Table 4). After washings, cell sorting was performed with FACSAria II (BD Bioscience).

For analysis of quiescent or G_0_ cells based on Hoechst (DNA content)/Pyronin Y (RNA content), single-cell suspensions of bones were prepared by collagenase digestion as described above. Cells were then stained using Hoechst 33342 (5 μg/mL for 45 minutes at 37°C in staining buffer); 1 μg/mL Pyronin Y was added, and cells were incubated for an additional 45 minutes prior to washing and flow cytometry.

### Isolation of supernatants from bone.

For ELISA and analysis of the impact of the bone secretome on cancer cells in vivo and in vitro, supernatant from mouse long bones was used. Dissected tibial bones were collected in ice-cold PBS and cleaned to remove the surrounding muscle tissue. The distal end of the tibial bones was chopped, and samples were placed in an 1.5 mL Eppendorf tube with the distal end facing the bottom of the tube. Bones were then centrifuged for 10 minutes at 4°C at maximum speed. After centrifugation, 100 μL ice-cold PBS was added, and samples were centrifuged again. 70 μL of the total supernatant accumulated at the bottom of the tube after centrifugation was collected. Pellet was discarded, and the supernatant was centrifuged again.

### ELISA.

Bmp4, Tgfb2, Thbs2, and Kitl levels in mouse bone supernatant/extracellular fluid were determined by ELISA kits (Sigma-Aldrich, Wuhan USCN Business Co. Ltd., and LifeSpan BioSciences Inc.) based on the manufacturer’s instructions.

### Statistical analysis.

All data are presented as mean ± SD. The significance of the difference in mean values was determined using 2-tailed Student’s *t* test, unless otherwise indicated. *P* < 0.05 was considered significant. Data presented in the figures are based on 3 independent experiments. *P* ≥ 0.05 was considered not significant. For analysis of the statistical significance of differences between more than 2 groups, we performed repeated-measures 1-way ANOVA tests with Tukey’s multiple-comparisons test or Dunnett’s multiple-comparisons test. All statistical analyses were performed using GraphPad Prism software. No randomization or blinding was used, and no animals were excluded from analysis. Sample sizes were selected on the basis of previous experiments. Several independent experiments were performed to guarantee reproducibility of findings.

### Study approval.

This investigation was performed in accordance with the Home Office Guidance on the Operation of the Animals (Scientific Procedures) Act 1986 (United Kingdom). Animals were maintained humanely in compliance with the *Principles of Laboratory Animal Care* formulated by the National Society for Medical Research and the *Guide for the Care and Use of Laboratory Animals* (National Academies Press, 2011). All animal protocols were approved by both the local University of Oxford or Imperial College London Animal Welfare and Ethical Review Board and by the UK Government Home Office (Animals Scientific Procedures Group).

## Author contributions

APK conceived the study and wrote the manuscript. AS, VV, RL, JC, SGR, SKR, and APK prepared, commented on, and revised the manuscript. AS, VV, PX, RL, JC, SGR, SKR, and APK performed experiments and analyzed the data.

## Supplementary Material

Supplemental data

Supplemental Table 3

Supplemental Table 4

Supplemental Table 5

Supplemental Table 6

Supplemental Table 7

Supplemental Table 8

## Version 1 07/11/2019

Electronic publication

## References

[1] Ramasamy SK, Kusumbe AP, Wang L, Adams RH (2014). Endothelial Notch activity promotes angiogenesis and osteogenesis in bone. Nature.

[2] Morrison SJ, Scadden DT (2014). The bone marrow niche for haematopoietic stem cells. Nature.

[3] Ramasamy SK, Kusumbe AP, Itkin T, Gur-Cohen S, Lapidot T, Adams RH (2016). Regulation of hematopoiesis and osteogenesis by blood vessel-derived signals. Annu Rev Cell Dev Biol.

[4] Wei Q, Frenette PS (2018). Niches for hematopoietic stem cells and their progeny. Immunity.

[5] Itkin T (2016). Distinct bone marrow blood vessels differentially regulate haematopoiesis. Nature.

[6] Rafii S, Butler JM, Ding BS (2016). Angiocrine functions of organ-specific endothelial cells. Nature.

[7] Romeo SG, Alawi KM, Rodrigues J, Singh A, Kusumbe AP, Ramasamy SK (2019). Endothelial proteolytic activity and interaction with non-resorbing osteoclasts mediate bone elongation. Nat Cell Biol.

[8] Kusumbe AP (2016). Age-dependent modulation of vascular niches for haematopoietic stem cells. Nature.

[9] Kusumbe AP, Ramasamy SK, Adams RH (2014). Coupling of angiogenesis and osteogenesis by a specific vessel subtype in bone. Nature.

[10] Flach J (2014). Replication stress is a potent driver of functional decline in ageing haematopoietic stem cells. Nature.

[11] Geiger H, de Haan G, Florian MC (2013). The ageing haematopoietic stem cell compartment. Nat Rev Immunol.

[12] Ho TT (2017). Autophagy maintains the metabolism and function of young and old stem cells. Nature.

[13] Guidi N (2017). Osteopontin attenuates aging-associated phenotypes of hematopoietic stem cells. EMBO J.

[14] Liu H, Xia X, Li B (2015). Mesenchymal stem cell aging: mechanisms and influences on skeletal and non-skeletal tissues. Exp Biol Med (Maywood).

[15] Ambrosi TH (2017). Adipocyte accumulation in the bone marrow during obesity and aging impairs stem cell-based hematopoietic and bone regeneration. Cell Stem Cell.

[16] Tokoyoda K, Hauser AE, Nakayama T, Radbruch A (2010). Organization of immunological memory by bone marrow stroma. Nat Rev Immunol.

[17] Cheung TH, Rando TA (2013). Molecular regulation of stem cell quiescence. Nat Rev Mol Cell Biol.

[18] Rossi DJ, Seita J, Czechowicz A, Bhattacharya D, Bryder D, Weissman IL (2007). Hematopoietic stem cell quiescence attenuates DNA damage response and permits DNA damage accumulation during aging. Cell Cycle.

[19] Sethe S, Scutt A, Stolzing A (2006). Aging of mesenchymal stem cells. Ageing Res Rev.

[20] Raymaekers K, Stegen S, van Gastel N, Carmeliet G (2015). The vasculature: a vessel for bone metastasis. Bonekey Rep.

[21] Ferreira A, Alho I, Casimiro S, Costa L (2015). Bone remodeling markers and bone metastases: from cancer research to clinical implications. Bonekey Rep.

[22] Coenegrachts L (2010). Anti-placental growth factor reduces bone metastasis by blocking tumor cell engraftment and osteoclast differentiation. Cancer Res.

[23] Mundy GR (2002). Metastasis to bone: causes, consequences and therapeutic opportunities. Nat Rev Cancer.

[24] Kusumbe AP (2016). Vascular niches for disseminated tumour cells in bone. J Bone Oncol.

[25] Psaila B, Lyden D (2009). The metastatic niche: adapting the foreign soil. Nat Rev Cancer.

[26] Suva LJ, Washam C, Nicholas RW, Griffin RJ (2011). Bone metastasis: mechanisms and therapeutic opportunities. Nat Rev Endocrinol.

[27] Passaro D (2017). Increased vascular permeability in the bone marrow microenvironment contributes to disease progression and drug response in acute myeloid leukemia. Cancer Cell.

[28] Quayle L, Ottewell PD, Holen I (2015). Bone metastasis: molecular mechanisms implicated in tumour cell dormancy in breast and prostate cancer. Curr Cancer Drug Targets.

[29] Ghajar CM (2013). The perivascular niche regulates breast tumour dormancy. Nat Cell Biol.

[30] Coleman RE (2011). Bone cancer in 2011: prevention and treatment of bone metastases. Nat Rev Clin Oncol.

[31] Mastro AM, Gay CV, Welch DR (2003). The skeleton as a unique environment for breast cancer cells. Clin Exp Metastasis.

[32] Sosa MS, Bragado P, Aguirre-Ghiso JA (2014). Mechanisms of disseminated cancer cell dormancy: an awakening field. Nat Rev Cancer.

[33] Maryanovich M (2018). Adrenergic nerve degeneration in bone marrow drives aging of the hematopoietic stem cell niche. Nature Medicine.

[34] Röcken M (2010). Early tumor dissemination, but late metastasis: insights into tumor dormancy. J Clin Invest.

[35] Cao Z (2014). Angiocrine factors deployed by tumor vascular niche induce B cell lymphoma invasiveness and chemoresistance. Cancer Cell.

[36] Recasens A, Munoz L (2019). Targeting cancer cell dormancy. Trends Pharmacol Sci.

[37] Wahlestedt M, Pronk CJ, Bryder D (2015). Concise review: hematopoietic stem cell aging and the prospects for rejuvenation. Stem Cells Transl Med.

[38] Pang WW (2011). Human bone marrow hematopoietic stem cells are increased in frequency and myeloid-biased with age. Proc Natl Acad Sci USA.

[39] Lindblom P (2003). Endothelial PDGF-B retention is required for proper investment of pericytes in the microvessel wall. Genes Dev.

[40] Nash DT (1990). Alpha-adrenergic blockers: mechanism of action, blood pressure control, and effects of lipoprotein metabolism. Clin Cardiol.

[41] Ramasamy SK (2016). Blood flow controls bone vascular function and osteogenesis. Nat Commun.

[42] Augustin HG, Koh GY (2017). Organotypic vasculature: from descriptive heterogeneity to functional pathophysiology. Science.

[43] Teichert M (2017). Pericyte-expressed Tie2 controls angiogenesis and vessel maturation. Nat Commun.

[44] Xian X (2006). Pericytes limit tumor cell metastasis. J Clin Invest.

[45] Carmeliet P, Jain RK (2000). Angiogenesis in cancer and other diseases. Nature.

[46] Maishi N, Hida K (2017). Tumor endothelial cells accelerate tumor metastasis. Cancer Sci.

[47] Kusumbe AP, Bapat SA (2009). Cancer stem cells and aneuploid populations within developing tumors are the major determinants of tumor dormancy. Cancer Res.

[48] Kim D, Pertea G, Trapnell C, Pimentel H, Kelley R, Salzberg SL (2013). TopHat2: accurate alignment of transcriptomes in the presence of insertions, deletions and gene fusions. Genome Biol.

[49] Anders S, Pyl PT, Huber W (2015). HTSeq — a Python framework to work with high-throughput sequencing data. Bioinformatics.

[50] Love MI, Huber W, Anders S (2014). Moderated estimation of fold change and dispersion for RNA-seq data with DESeq2. Genome Biol.

[51] Hon J, Martínek T, Rajdl K, Lexa M (2013). Triplex: an R/Bioconductor package for identification and visualization of potential intramolecular triplex patterns in DNA sequences. Bioinformatics.

