# Supplementary material for: Angiocrine signals regulate quiescence and therapy resistance in bone metastasis
Source: JCI Insight. 2019 Jul 11;4(13):e125679. doi: 10.1172/jci.insight.125679 (PMC6629249; doi:10.1172/jci.insight.125679)
Supplement: Supplemental data [file jciinsight-4-125679-s016.pdf]

Supplementary Figures

Supplementary Figure. 1: Age-dependent changes in cancer cell expansion, quiescence and cytokine expression.

A, Bar graph shows flow cytometric quantification of MDA-MB-231-GFP cells in the single cell suspension of young and aged mice bones. The data represents mean ± s.d. (*n*=5 replicates), P value, and two-tailed unpaired *t*-test.

B, Bar graph shows flow cytometric quantification of ZR-75-1-GFP cells in the single cell suspension of young and aged mice bones. The data represents mean ± s.d. (*n*=5 replicates), P value, and two-tailed unpaired *t*-test.

C, Bar graph demonstrates quantification of quiescent (G0) MDA-MB-231-GFP cells in bones of young and aged mice after intra-tibial injections. The data represents mean ± s.d. (*n*=5 replicates), P value, and two-tailed unpaired *t*-test.

D, Bar graph demonstrates quantification of quiescent (G0) ZR-75-1 cells in bones of young and aged mice after intra-tibial injections. The data represents mean ± s.d. (*n*=5 replicates), P value, and two-tailed unpaired *t*-test.

E, Bar graph showing qPCR analysis of *Il6* and *Il1b* expression (normalized to *Actb*) in aged relative to young tibiae. The data represent mean ± s.d. (*n*=8 replicates), P values, and two-tailed unpaired *t*-tests. ***: P <0.001; ****: P <0.0001.

Supplementary Figure. 2: Secreted factors from the BM microenvironment regulate bone metastatic cancer cells.

A, Bar graph showing FACS quantifications of proliferating MCF-7-GFP cells in culture by Ki67 immunostaining in PBS and aged BM secretome (aged-BM-sec) treated cells. The data represents mean ± s.d. (*n*=7 replicates), P value, and two-tailed unpaired *t*-test.

B, Bar graph shows flow cytometric quantification of MCF7-GFP cells in the single cell suspension of tibiae from young mice. The data represents mean ± s.d. (*n*=7 replicates), P value, and two-tailed unpaired *t*-test.

C, Bar graph shows flow cytometric quantifications of $Ki67^+$ MCF7-GFP cells in sham (PBS) and aged-BM-sec injected young murine tibiae. The data represents mean ± s.d. (*n*=7 replicates), P value, and two-tailed unpaired *t*-test. ****: P <0.0001.

Supplementary Figure. 3: Radiation and chemotherapy induced alterations in quiescence inducing secreted factors.

A, Venn diagram displaying the overlapping of the significantly up/down regulated genes between radiation treated and chemotherapy treated samples of young mice bones compared to control mouse bones as identified by RNA-seq with FDR-adjusted p-value < 0.01 and absolute log2 fold change ± 1.

B-C, Heat map showing the most significant known cellular quiescence inducing secreted factors, differentially expressed in bones from young radiation treated or carboplatin treated (as indicated in the figure) versus control young mice. The color code indicating the row mean subtracted normalized log2(CPM) expression values.

D-F, Bar graph showing qPCR analysis of *Bmp4, Bmp6, Bmp7, Kitl, Tgfb2, and Thbs2* expression (normalized to *Actb*) by young radiation treated, carboplatin treated or cisplatin treated (as indicated in figure) tibiae relative to young control tibiae. The data represent mean ± s.d. (*n*=5 replicates), P values, and two-tailed unpaired *t*-tests.

G, ELISA analyses of Bmp4 in BM supernatants of femurs from radiation treated and chemotherapy/cisplatin treated bones from young and aged mice. The data represent mean ± s.d. (*n*=6 replicates), P values, and two-tailed unpaired *t*-tests.

H, ELISA analyses of Tgfb2 in BM supernatants of femurs from radiation treated and chemotherapy/cisplatin treated bones from young and aged mice. The data represent mean ± s.d. (n=5 replicates), P values, and two-tailed unpaired *t*-tests.

I, ELISA analyses of Thbs2 in BM supernatants of femurs from radiation treated and

chemotherapy/cisplatin treated bones from young and aged mice. The data represent mean ± s.d. (n=5 replicates), P values, and two-tailed unpaired *t*-tests.

J, ELISA analyses of Kitl in BM supernatants of femurs from radiation treated and chemotherapy/cisplatin treated bones from young and aged mice. The data represent mean ± s.d. (n=6 replicates), P values, and two-tailed unpaired *t*-tests.

K, Bar graph showing qPCR analysis of *Bmp4, Bmp6, Bmp7, Kitl, Tgfb2, and Thbs2* expression (normalized to *Actb*) by aged radiation treated (as indicated in figure) tibiae relative to aged control tibiae. The data represent mean ± s.d. (*n*=5 replicates), P values, and two-tailed unpaired *t*-tests.

L, Bar graph showing qPCR analysis of *Bmp4, Bmp6, Bmp7, Kitl, Tgfb2, and Thbs2* expression (normalized to *Actb*) by carboplatin treated aged tibiae relative to control aged tibiae. The data represent mean ± s.d. (*n*=5 replicates), P values, and two-tailed unpaired *t*-tests.

*: P <0.05; **: P <0.01; ***: P <0.001; ****: P <0.0001.

Supplementary Figure. 4: Radiation and chemotherapy induced alterations in cytokine expressions.

A, Scatterplot showing average normalized log2(CPM) control bone in y-axis and average normalized log2(CPM) radiation treated young mouse bones in x axis. Each black dot represents cytokines' gene name. Red line depicts slop and intercept.

B, Scatterplot showing y-axis mean normalized log2(CPM) of control bones and x-axis mean normalized log2(CPM) chemotherapy treated young mouse bones. Black dots represent the known cytokine genes. Red line indicates the slop and intercept dividing the cytokines' expression in control sample along the y-axis to chemotherapy treated cytokines expression in x-axis.

C, Bar graph showing qPCR analysis of *Il6* and *Il1b* expression (normalized to *Actb*) by radiation

treated young tibiae relative to control young tibiae. The data represent mean ± s.d. (*n*=8 replicates), P values, and two-tailed unpaired *t*-tests.

D, Bar graph showing qPCR analysis of *Il6* and *Il1b* expression (normalized to *Actb*) by carboplatin treated young tibiae relative to control young tibiae. The data represent mean ± s.d. (*n*=8 replicates), P values, and two-tailed unpaired *t*-tests.

E, Bar graph showing qPCR analysis of *Il6* and *Il1b* expression (normalized to *Actb*) by cisplatin treated young tibiae relative to control young tibiae. The data represent mean ± s.d. (*n*=8 replicates), P values, and two-tailed unpaired *t*-tests. ****: P <0.0001.

Supplementary Figure. 5: Age-dependedent changes in cell surface marker expressions in BM

A, Bar graph showing qPCR analysis of *Bcam, Cspg4, Emcn, Pdgfra, Pdgfrb and Pecam1* expression (normalized to *Actb*) by in young versus aged tibiae. The data represent mean ± s.d. (*n*=8 replicates), P values, and two-tailed unpaired *t*-tests.

B, Bar graph showing qPCR analysis of *Lepr* expression (normalized to *Actb*) in young versus aged tibiae. The data represents mean ± s.d. (*n*=7 replicates), P value, and two-tailed unpaired *t*-test. ****: P <0.0001, ns: not significant.

Supplementary Figure. 6: Chemotherapy induced expansion of pericytes in bone

A, Representative tile scan confocal images showing PDGFRβ + α-SMA (green) and Endomucin/Emcn (red) immunostaining on thick tibial sections from control and carboplatin treated young mice. Nuclei, TO-PRO-3 (blue). Metaphysis (mp); diaphysis (dp); growth plate (gp). Scale bars: 400 μm. Representative images are derived based on three independent experiments. ns: not significant, UD: undetected.

B, Bar graph showing qPCR analysis of *Bmp4, Bmp6, Bmp7, Thbs2 and Kitl* expression (normalized to *Actb*) in PDGFRβ^+ pericytes isolated from young and aged bones. The data represent mean ± s.d. (*n*=3 replicates) and two-tailed unpaired *t*-tests. Scale bars: 400 μm.

Supplementary Figure. 7: Radiation induced expansion of pericytes is bone-specific.

A, Representative confocal images showing PDGFRβ (green) immunostaining on thick sections from heart, kidney, liver and spleen in control and radiation treated young mice. Nuclei, TO-PRO-3 (blue). Note: No remarkable pericytes expansion upon radiation treatment in these organs. Scale bars: 50 μm. Representative images are derived based on three independent experiments.

B, Bar graph showing qPCR analysis of *Pdgfrb* expression (normalized to *Actb*) in young versus aged tibiae. The data represents mean ± s.d. (*n*=5 replicates), P value, and two-tailed unpaired *t*-test.

Supplementary Figure. 8: Age-dependent decline in cellular quiescence inducing factors is bone-specific.

A-F, Bar graphs showing qPCR analysis of *Bmp4, Bmp6, Bmp7, Kitl, Tgfb2 and Thbs2* expression (normalized to *Actb*) in young versus aged liver, kidney, heart, lung, spleen and brain (as indicated in the figure). The data represent mean ± s.d. (*n*=5 replicates), P values, and two-tailed unpaired *t*-tests. *: P <0.05; **: P <0.01; ***: P <0.001; ****: P <0.0001, ns: not significant.

Supplementary Figure. 9: Blood flow induced changes in the BM microenvironment.

A, ELISA analysis of PDGF-BB in BM supernatants of femurs from radiation treated and chemotherapy/cisplatin treated bones from young and aged mice. The data represent mean ± s.d. (*n*=7 replicates), P values, and two-tailed unpaired *t*-tests.

B, Bar graph showing qPCR analysis of *Pdgfb* expression (normalized to *Actb*) in control (indicated by green bars) versus radiation (indicated by red bars) liver, lung, kidney, heart and brain from young mice. The data represent mean ± s.d. (*n*=5 replicates), P values, and two-tailed unpaired *t*-tests.

C, Bar graph showing qPCR analysis of *Pdgfb* expression (normalized to *Actb*) in sorted endothelial subsets, type H, type L and arterial ECs. Data represents mean±s.d. (*n*=7 replicates) P values, one-way ANOVA by Tukey's multiple comparison post-hoc test.

D, Bar graph showing quantification of type H ECs by flow cytometry in femurs from young Clonidine treated and young control mice. The data represent mean ± s.d. (*n*=7 replicates), P values, and two-tailed unpaired *t*-tests.

E, The bar graphs showing FACS quantification of PDGFRβ+ ($CD31^-/CD45^-/Ter119^-$) perivascular cells in young Clonidine treated versus young control femurs. The data represents the mean ± s.d. (*n*=7 replicates), P value, and two-tailed unpaired *t*-test. ***: P <0.001; ****: P <0.0001, ns: not significant.

Supplementary Figure. 10: BM microenvironment regulates stem and cancer cell quiescence.

A, Schematic illustration showing age-dependent changes in stem and cancer cell quiescence.

B, Radiation and chemotherapy induced changes in BM microenvironment and cancer cell quiescence. Note: Expansion of pericytes upon radiation or chemotherapy treatments, and targeting of pericytes renders cancer cells susceptible to radiation and chemotherapy.

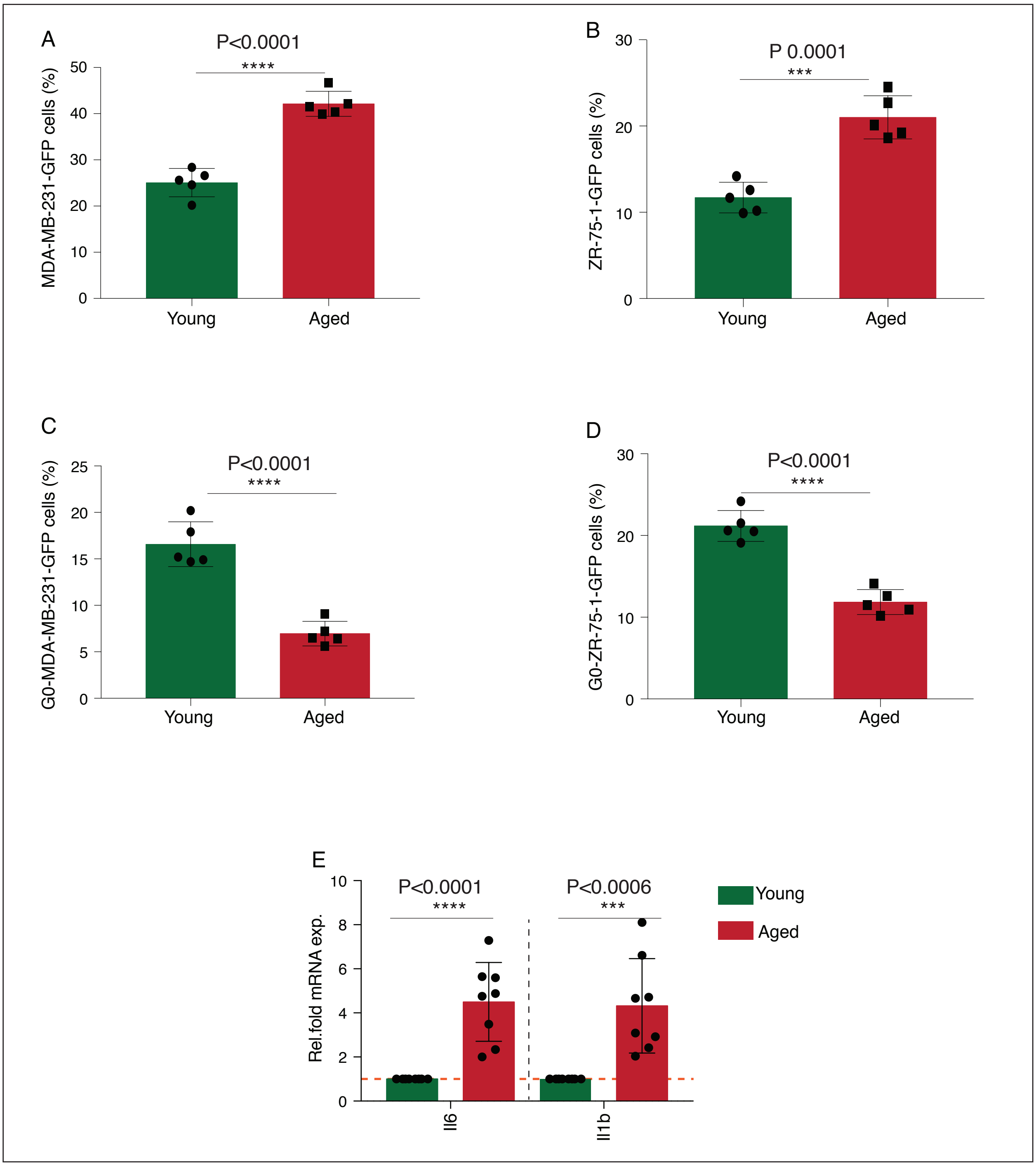

Supplementary Figure. 1

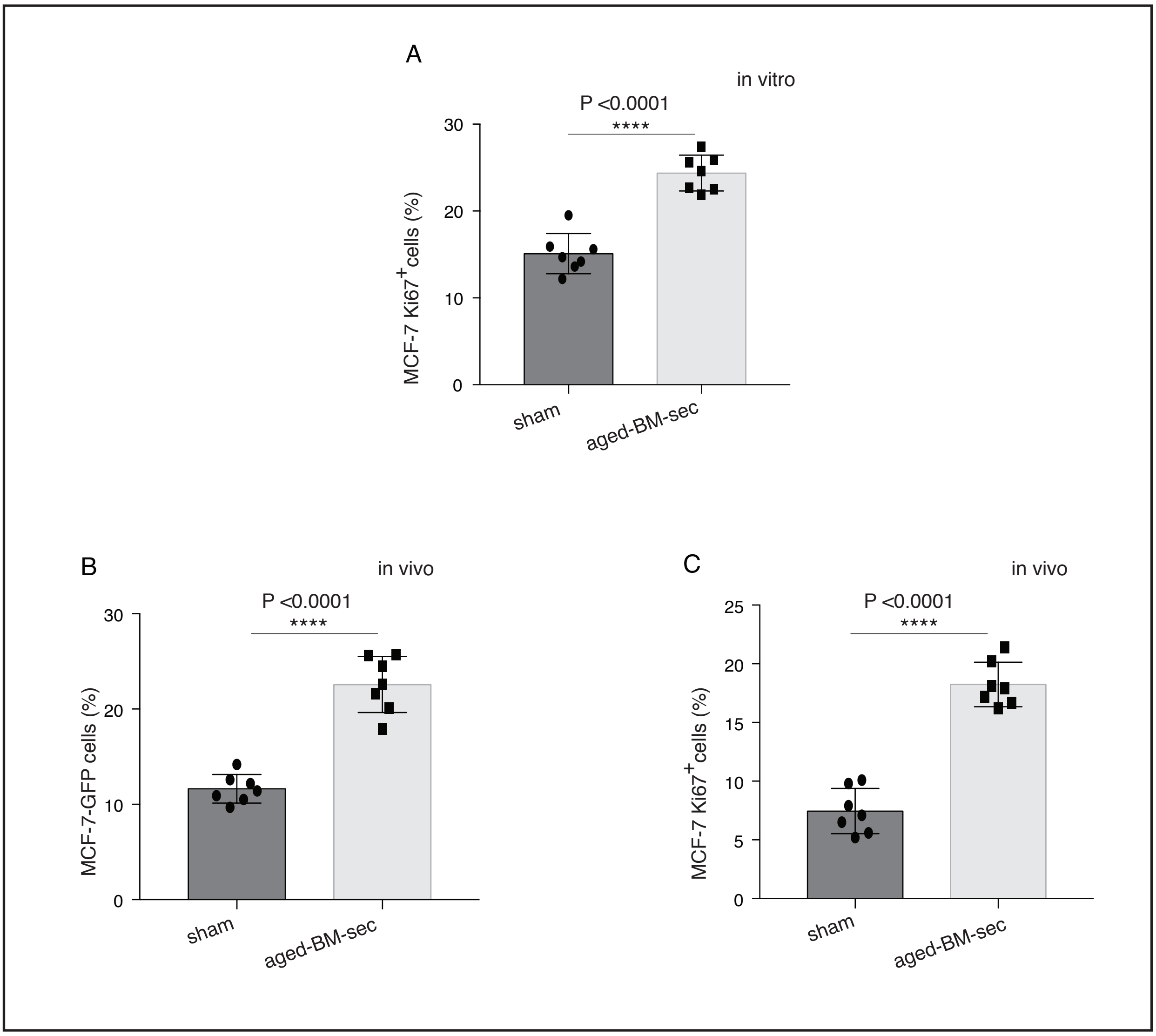

Supplementary Figure. 2

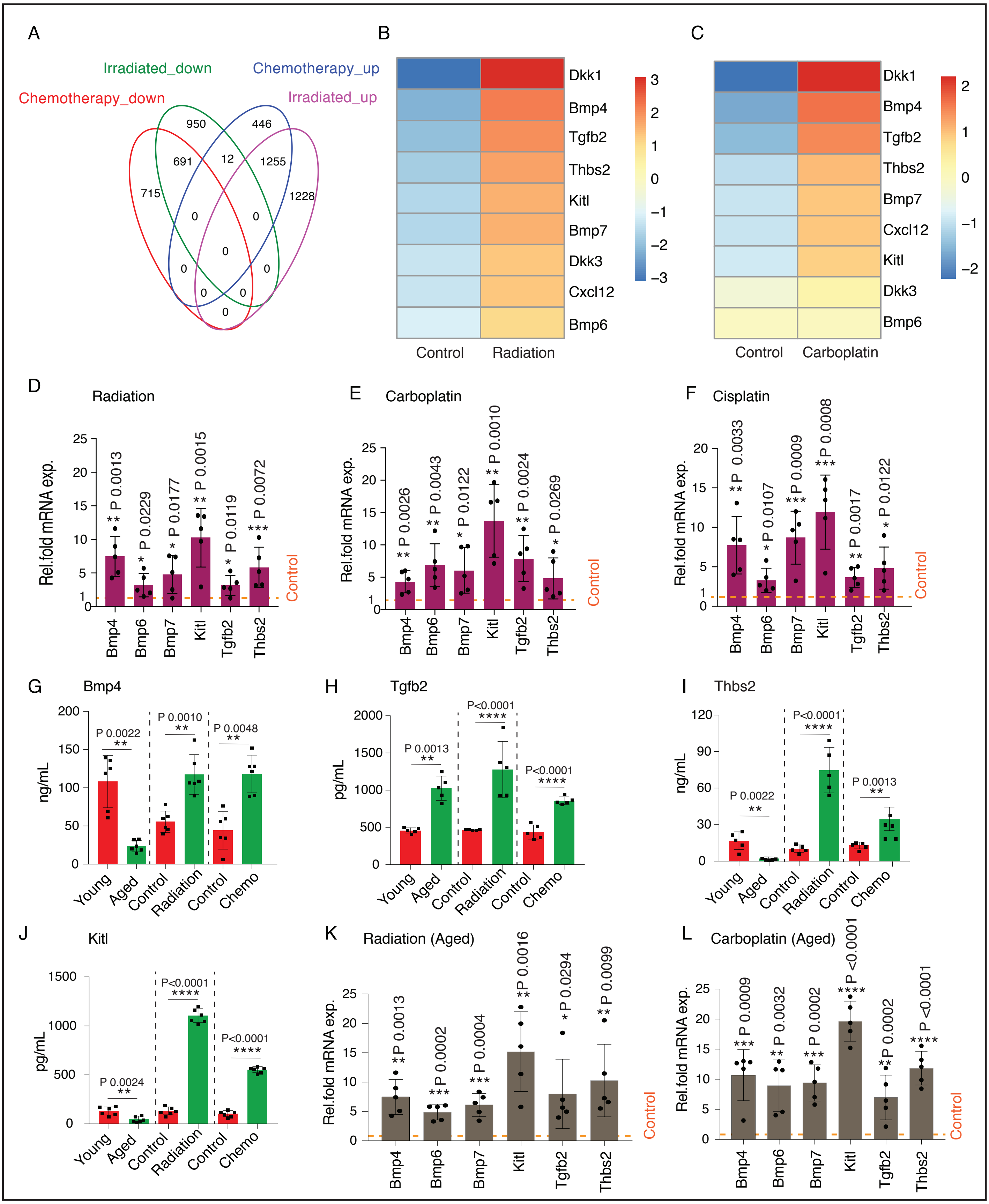

Supplementary Figure. 3

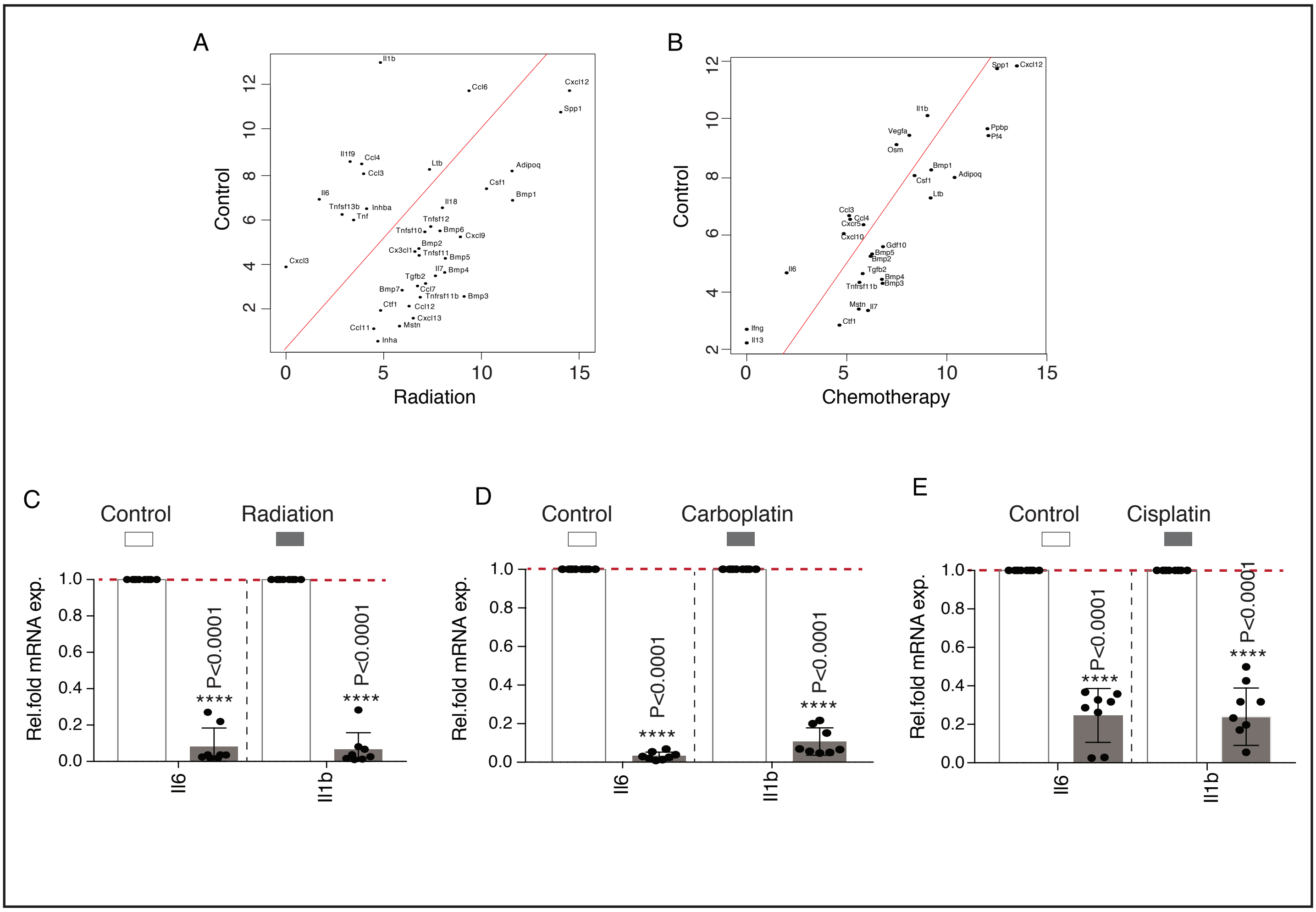

Supplementary Figure. 4

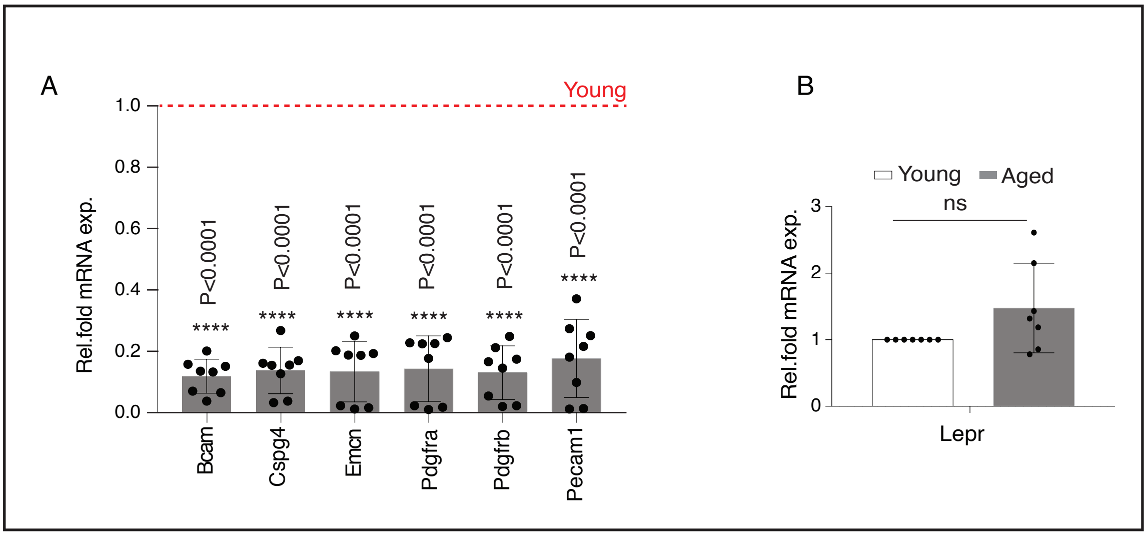

Supplementary Figure. 5

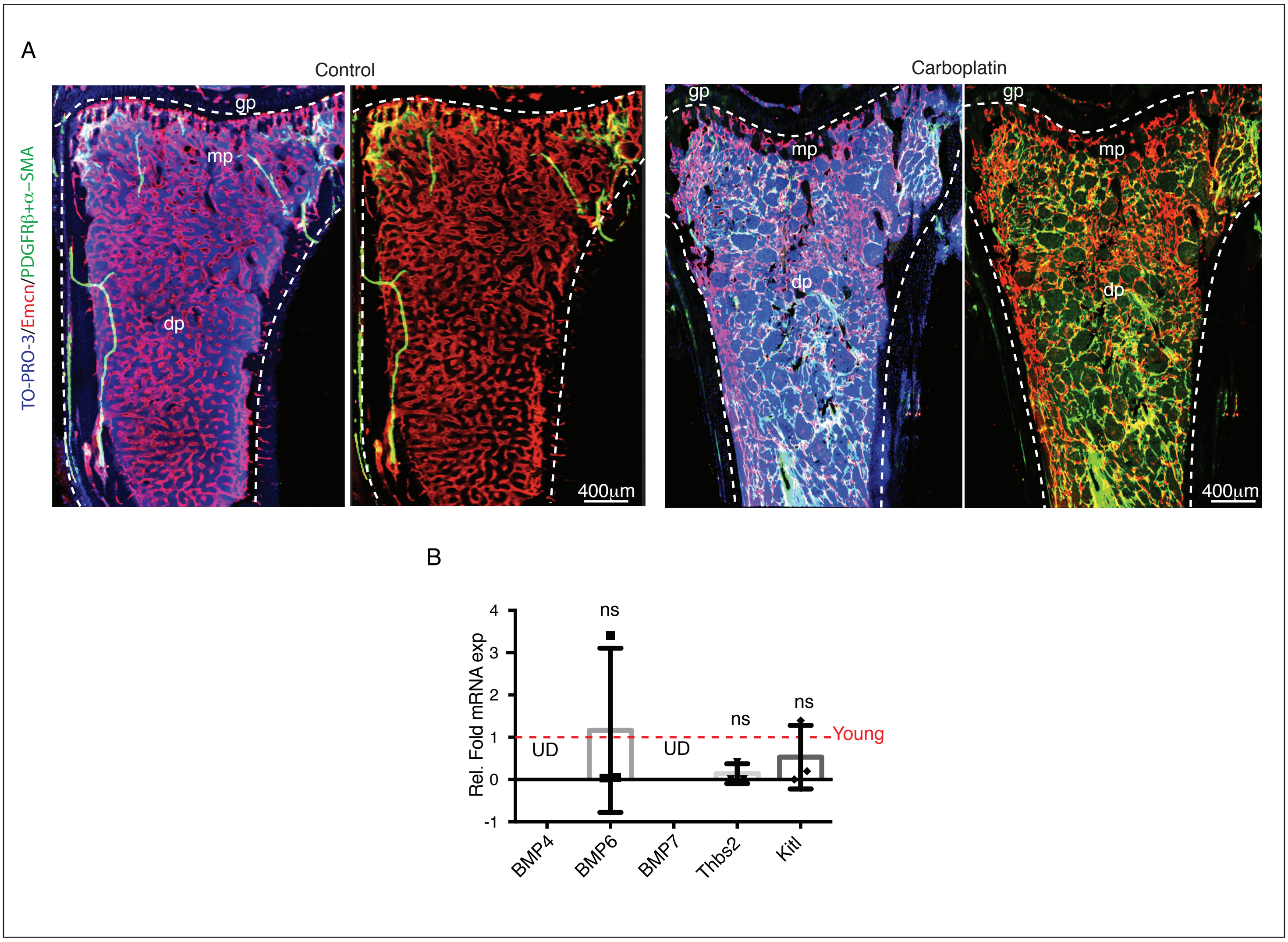

Supplementary Figure. 6

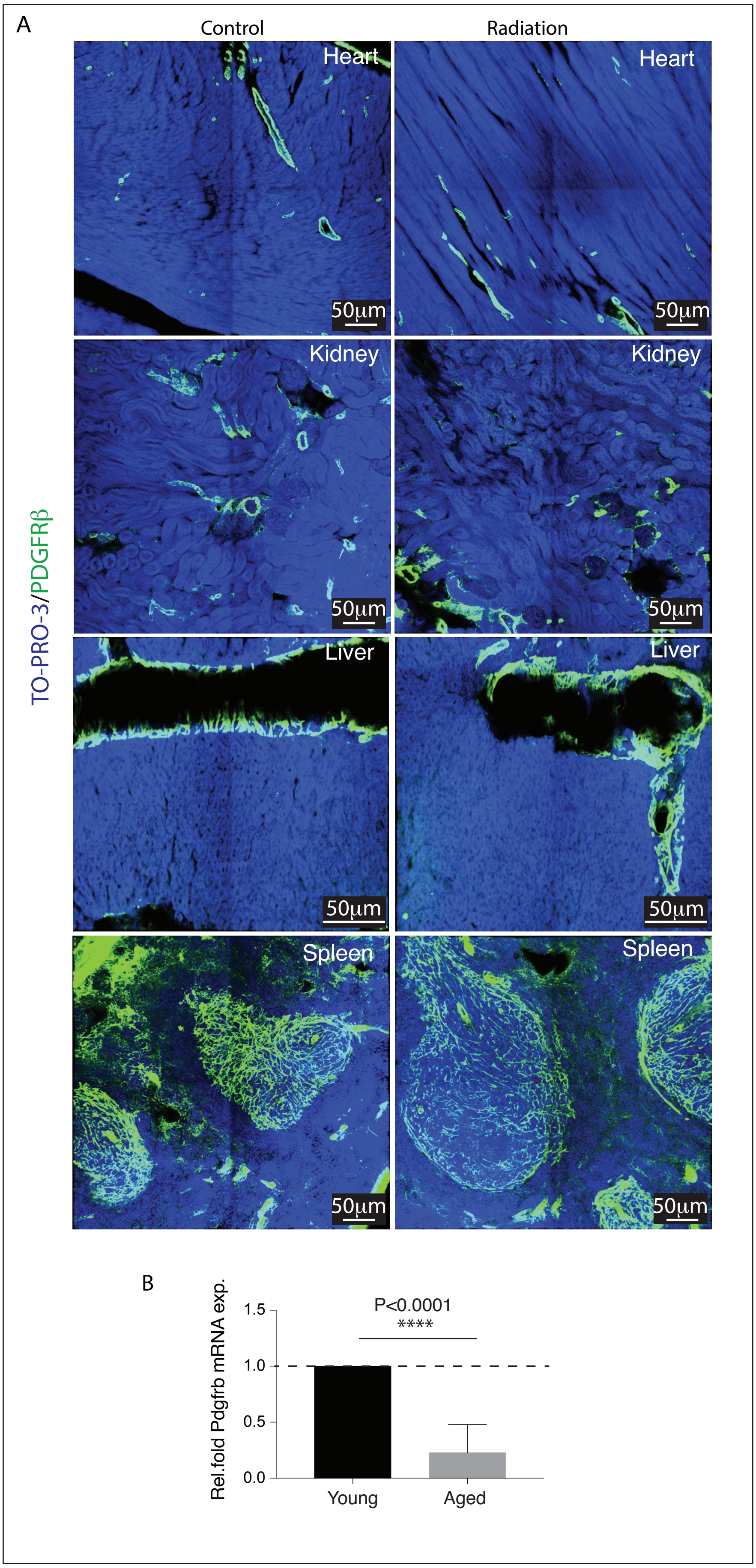

Supplementary Figure. 7

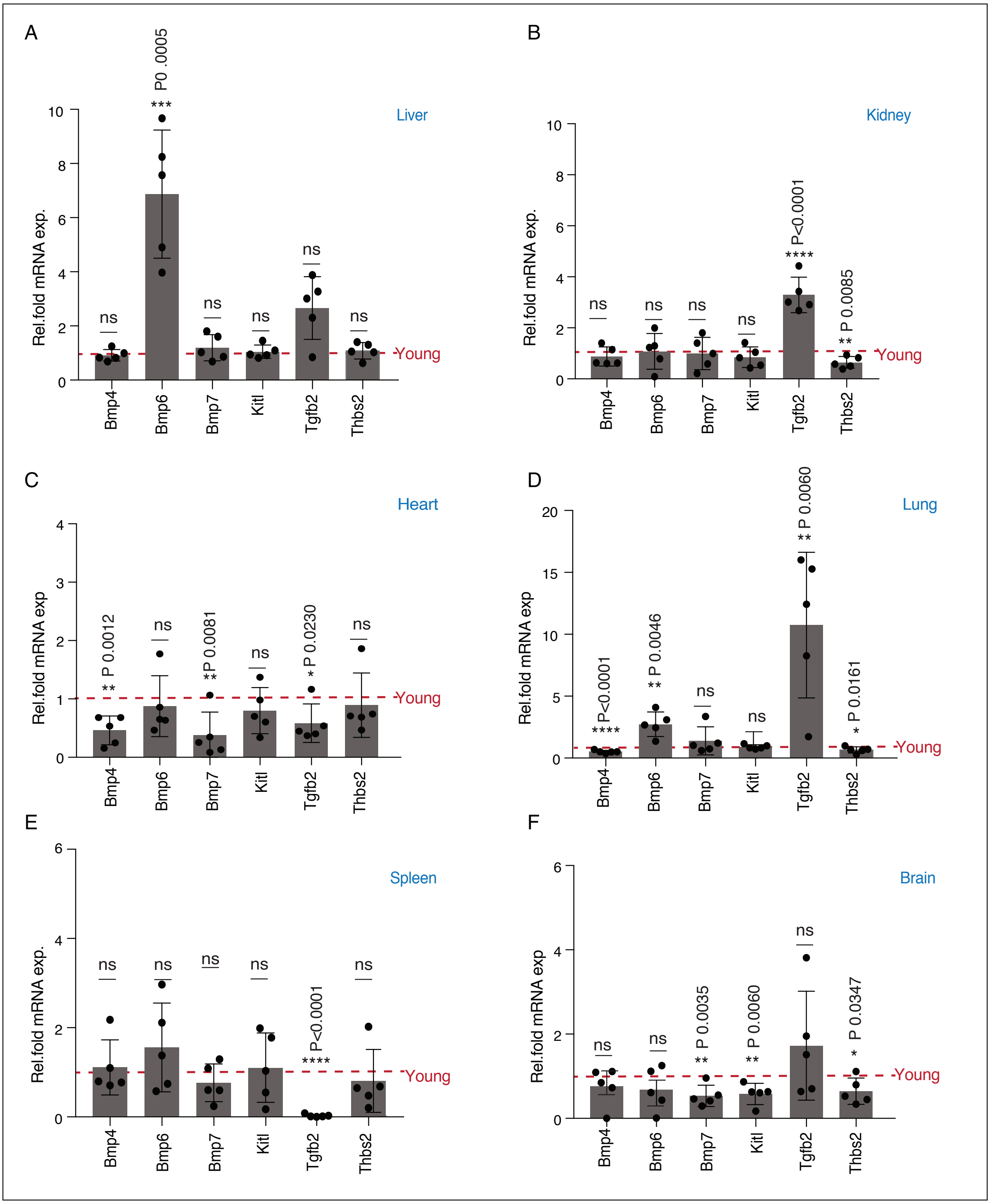

Supplementary Figure. 8

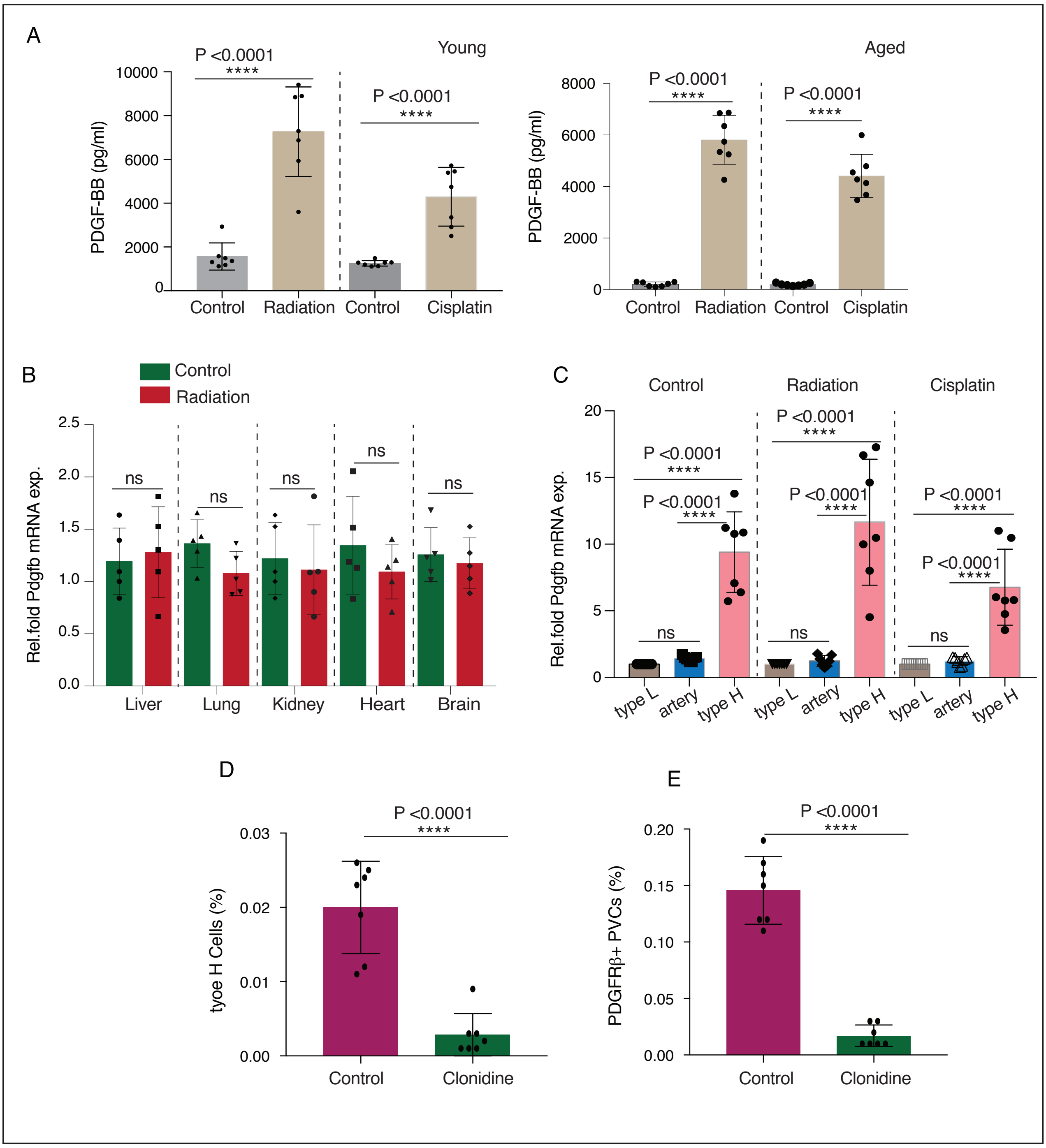

Supplementary Figure. 9

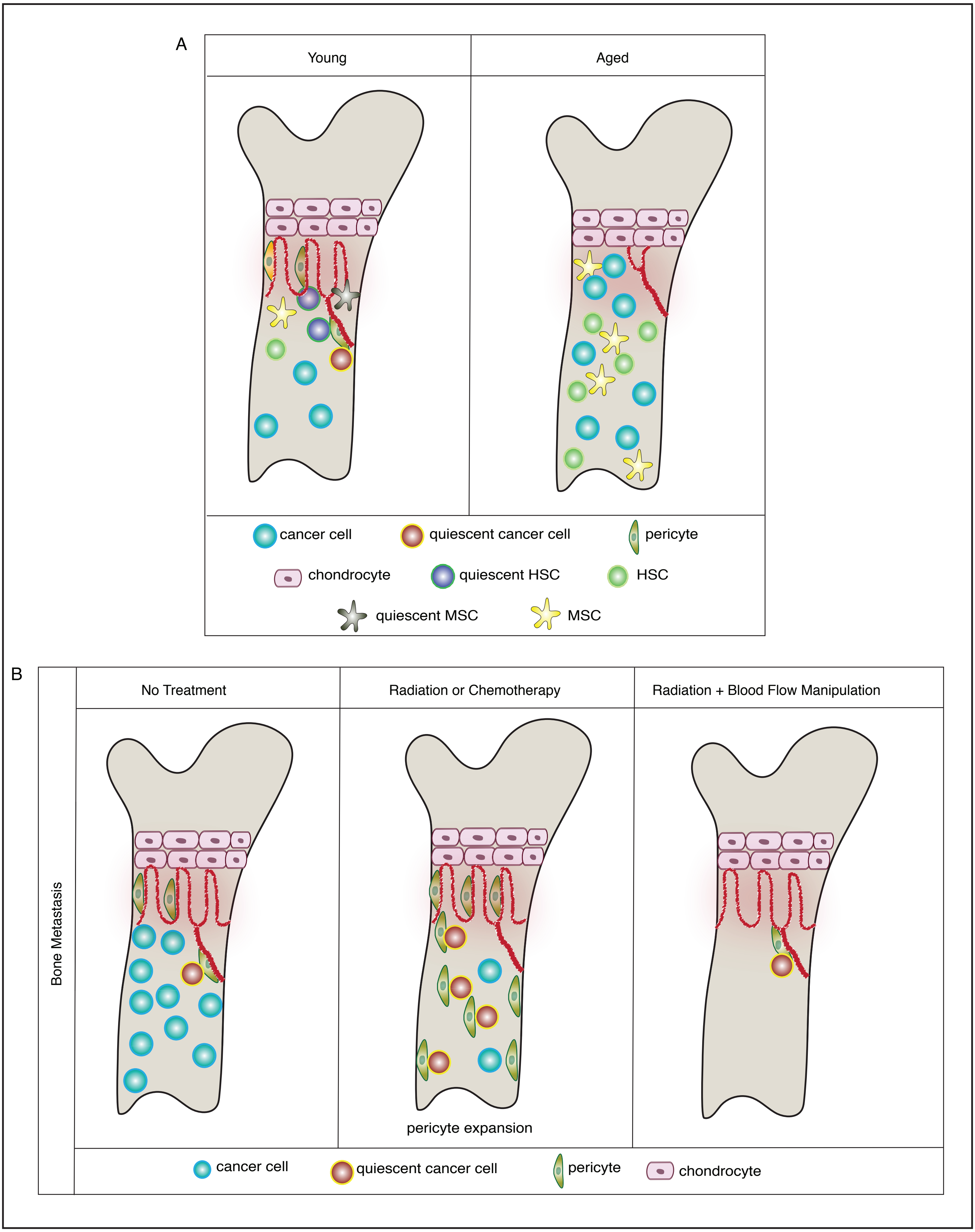

Supplementary Figure. 10

Supplementary Table 1: List of the primary antibodies used for used for FACS and immunostaining.

	Primary Antibodies	Clone/Cat. No	Company	Application	Conjugation
1	Endomucin	V.7C7/ sc-65495	Santa Cruz	FACS, cell sorting	Unconjugated
2	CD31/PECAM-1	FAB3628G	R&D Systems	FACS	Alexa Fluor 488
3	CD31/PECAM-1	FAB3628A	R&D Systems	FACS	APC
4	Pecam1	MEC 13.3/553370	BD Pharmingen	FACS	Unconjugated
5	PDGFRb	Y92 /ab32570	Abcam	IHC/FACS	Unconjugated
6	Alpha smooth muscle actin	1A4/C6198	Sigma-Aldrich	IHC	Cy3
7	GFP	A21311	Invitrogen	IHC/FACS	Alexa Fluor 488
8	CD45	30-F11/553077	BD Pharmingen	FACS	Biotin
9	CD117(c-kit)	2B8/105814	BioLegend	FACS	PE/Cy7
10	CD150(SLAM)	TC15-12F12.2/115904	BioLegend	FACS	PE
11	Ly-6G (Gr-1)	RB6-8C5/108412	BioLegend	FACS	Biotin
12	Ki-67	16A8/652409	BioLegend	FACS	FITC
13	CD48	HM48-1/103432	BioLegend	FACS	APC
14	CD45R/B220	RA3-6B2/103212	BioLegend	FACS	APC
15	CD45	30-F11/103105	BioLegend	FACS	PE
16	CD45	30-F11/103101	BioLegend	FACS	Unconjugated
17	CD45	30-F11/35-0451	TONBO Biosciences	FACS	FITC
18	Ly-6A/E (Sca-1)	D7/108127	BioLegend	FACS	Brilliant Violet 421
19	Ly-6A/E (Sca-1)	D7/108120	BioLegend	FACS	Pacific Blue
20	Ly-6A/E (Sca-1)	E13-161.7/122505	BioLegend	FACS	FITC
21	CD31	390/102405	BioLegend	FACS	FITC
22	CD31	MEC13.3/102509	BioLegend	FACS	APC
23	CD31/PECAM-1	390/102401	BioLegend	FACS	Unconjugated
24	TER-119	559971	BD Pharmingen	FACS	Biotin
25	TER-119	TER-119/30-5921	TONBO Biosciences	FACS	Biotin
26	TER-119	TER-119/116201	BioLegend	FACS	Unconjugated
27	TER-119/Erythroid cells	TER-119/116212	BioLegend	FACS	APC
28	TER-119	TER-119/50-5921-U025	TONBO Biosciences	FACS	PE
29	CD140b	APB5/136007	BioLegend	FACS	APC
30	CD140a	APA5/135907	BioLegend	FACS	APC
31	CD140a	APA5/11-1401-82	eBioscience	FACS	FITC
32	CD11b	M1/70/101212	BioLegend	FACS	APC
33	CD11a	M17/4/101120	BioLegend	FACS	APC
34	CD4	RM4-5/100516	BioLegend	FACS	APC
35	CD8a	53-6.7/100712	BioLegend	FACS	APC
36	RANKL	R12-31/14-6612-82	ThermoFishes Scientific	Cell sorting	Unconjugated
37	Bcam1	AF8299	R&D Systems	Cell Sorting	Unconjugated

Supplementary Table 2: List of primers used for qPCR. Gene name, sequence from 5'-3' and product size are listed for each primer used.

	Gene	Gene abbreviation	Sequence (5'-3')	Product Size (bp)
1	*bone morphogenetic protein 4*	*BMP4*	*F: CCTGGTAACCGAATGCTGAT*	*250*
			R: AGCCGGTAAAGATCCCTCAT	
2	*bone morphogenetic protein 6*	*BMP6*	*F: TTCTTCAAGGTGAGCGAGG*	*236*
			R: TAGTTGGCAGCGTAGCCTTT	
3	*bone morphogenetic protein 7*	*BMP7*	*F: GGGCTTACAGCTCTCTGTGG*	*297*
			R: AGGTCTCGGAAGCTGACGTA	
4	*mus musculus kit ligand*	*Kitl*	*F: TCATGGTGCACCGTATCCTA*	*170*
			R: CCTTGGCATGTTCTTCCACT	
5	*transforming growth factor, beta 2*	*Tgfb2*	*F: GCTCCAATTCTTTCCCCTTC*	*272*
			R: CCCACCCATATGCTAACACC	
6	*thrombospondin 2*	*Thbs2*	*F: GAACCAGCTGAGCAAGAACC*	*212*
			R: CAGGTGAGCAGGTGATCTGA	
8	*interleukin 6*	*IL6*	*F: GTTCTCTGGGAAATCGTGGA*	*339*
			R: GGAAATTGGGGTAGGAAGGA	
9	*interleukin 1 beta*	*IL1B*	*F: CAGGCAGGCAGTATCACTCA*	*350*
			R: AGGCCACAGGTATTTTGTCG	
10	*platelet derived growth factor, B polypeptide*	*Pdgfb*	*F: GCGAGCGAGTGGGTAGATAG*	*347*
			R: GCTAAAGGCGTGTTCCTCTG	
11	*basal cell adhesion molecule*	*Bcam*	*F: AGCTCGCTGATGGTGAAAGT*	*385*
			R: GTCGTCAGCCTCAACACTCA	
12	*chondroitin sulfate proteoglycan 4*	*Cspg4*	*F: GCACGATGACTCTGAGACCA*	*223*
			R: AGCATCGCTGAAGGCTACAT	
13	*endomucin*	*Emcn*	*F: CAGTGAAGCCACTGAGACCA*	*247*
			R: ACGTCACTTTTGGTCGTTCC	
14	*platelet derived growth factor receptor, alpha polypeptide*	*Pdgfra*	*F: GGGGAGAGTGAAGTGAGCTG*	*347*
			R: CTCCCGTTATTGTGCAAGGT	
15	*platelet derived growth factor receptor, alpha polypeptide*	*Pdgfrb*	*F: CCGGAACAAACACACCTTCT*	*313*
			R: GAGCACTGGTGAGTCGTTGA	
16	*platelet/endothelial cell adhesion molecule 1*	*Pecam1*	*F: GCCTGGAGAGGTTGTCAGAG*	*357*
			R: GGTGCTGAGACCTGCTTTTC	
17	*C-X-C Motif Chemokine Ligand 12*	*Cxcl12*	*F: AGTAGTGGCTCCCCAGGTTT*	*250*
			R:GAGACAGTCTTGCGGACACA	
18	*leptin receptor*	*Leptr*	*F:GGACACAGGTGGGACACTCT*	*249*
			R:CCCCACAGCACATTTTTCTT	
19	*beta actin*	*Actinb*	*F: TGTTACCAACTGGGACGACA*	*392*
			R:TCTCAGCTGTGGTGGTGAAG	
